# Environment-dependent striatal gene expression in the BACHD rat model for Huntington disease

**DOI:** 10.1038/s41598-018-24243-z

**Published:** 2018-04-11

**Authors:** Arianna Novati, Thomas Hentrich, Zinah Wassouf, Jonasz J. Weber, Libo Yu-Taeger, Nicole Déglon, Huu Phuc Nguyen, Julia M. Schulze-Hentrich

**Affiliations:** 1 Institute of Medical Genetics and Applied Genomics, University of Tübingen, Tübingen, Germany; 2 Department of Clinical Neurosciences (DNC), Lausanne University Hospital (CHUV), Lausanne, Switzerland; 3 Department of Human Genetics, Ruhr-University Bochum, Bochum, Germany

## Abstract

Huntington disease (HD) is an autosomal dominant neurodegenerative disorder caused by a mutation in the huntingtin (*HTT*) gene which results in progressive neurodegeneration in the striatum, cortex, and eventually most brain areas. Despite being a monogenic disorder, environmental factors influence HD characteristics. Both human and mouse studies suggest that mutant *HTT* (*mHTT*) leads to gene expression changes that harbor potential to be modulated by the environment. Yet, the underlying mechanisms integrating environmental cues into the gene regulatory program have remained largely unclear. To better understand gene-environment interactions in the context of *mHTT*, we employed RNA-seq to examine effects of maternal separation (MS) and environmental enrichment (EE) on striatal gene expression during development of BACHD rats. We integrated our results with striatal consensus modules defined on *HTT*-CAG length and age-dependent co-expression gene networks to relate the environmental factors with disease progression. While *mHTT* was the main determinant of expression changes, both MS and EE were capable of modulating these disturbances, resulting in distinctive and in several cases opposing effects of MS and EE on consensus modules. This bivalent response to maternal separation and environmental enrichment may aid in explaining their distinct effects observed on disease phenotypes in animal models of HD and related neurodegenerative disorders.

## Introduction

Huntington disease (HD) is an autosomal dominantly inherited neurodegenerative disorder caused by a polyglutamine expansion in exon 1 of the huntingtin gene (*HTT*)^1^. The mutation results in progressive neurodegeneration throughout the brain with strongest damages in the striatum which shows marked atrophy and loss of medium spiny neurons^2–4^. As a consequence, HD patients are burdened with motor deficits, cognitive impairments, and neuropsychiatric symptoms^1^.

The pathomechanical trajectory between mutant *HTT* (*mHTT*) and brain damage seems highly complex and different cellular functions have been implicated in mediating the toxicity. Previous studies in cell and animal models as well as human brains show that accumulation of *mHTT* leads to disturbed expression of genes associated with different neuronal functions^5,6^. Recently, these disturbances have been further explored in different HD mouse models carrying either fragment or full-length *mHTT*^7–10^, with respect to age and CAG length^11^, and also in BACHD (bacterial artificial chromosome HD) rats^12^.

Adding to the complexity, HD and related neurodegenerative disorders including Alzheimer disease, Parkinson’s disease, and Amyotrophic lateral sclerosis have been associated with environmental factors that bivalently correlate with disease risk and characteristics^13–16^. Previous studies in animal models showed positive effects on HD-like phenotypes through enriched housing, cognitive enrichment, and exercise. Specifically, enriched environments caused expression changes of distinct genes^17^, influenced markers of brain pathology^9,18–21^, altered behavioural^19,22–25^ as well as other parameters such as survival rate and body weight^26^. On the other hand, worsening of disease phenotypes in mouse models of HD was observed for various stressors. While acute stress led to increased depressive-like behaviour^19^ and impaired memory acquisition^25^, chronic stress affected olfactory sensitivity^27^ and accelerated memory deficits^28^. Despite the increasing body of evidence that environmental effects influence HD phenotypes in animal models, little is known about how these environmental cues are integrated into the gene regulatory program.

Towards a better understanding of these mechanisms, we examined interactions between environment and genotype in BACHD rats expressing full-length human *mHTT* and exhibiting motor impairments, cognitive deficits, emotional changes, and characteristic neuropathology of HD^29–32^. Given the greater sensitivity of the brain to environmental changes and challenges during development with known consequences for later stages in life^33–35^, we manipulated the rearing environment of BACHD rats in early life through maternal separation and environmental enrichment, respectively. While one group of rat pups was repeatedly separated from their mothers during the first 14 days of life to implement the stress paradigm, the other group was housed in the enriched environment from weaning till adulthood. As both environmental conditions are known to affect brain function and induce gene expression changes in wildtype animals^33–36^, subsequent profiling of the striatal transcriptome of wildtype and BACHD rats using RNA-seq enabled comprehensive analyses of gene expression changes in the context of *mHTT* and their modulations through the respective environmental paradigm.

## Results

### Full-length mutant *HTT* disturbed striatal gene expression depending on the environment

In order to assess the impact of environmental conditions on gene expression in the context of *mHTT*, we used a BAC transgenic rat model that expresses the full-length human *mHTT* gene with 97 polyQ repeats (BACHD)^29^ and exposed groups of animals (n=6 each) to either a standard environment (SE), enriched environment (EE), or maternal separation (MS) before profiling their striatal transcriptome using deep-sequencing of polyA-enriched RNA.

The enriched environment was set-up as a combination of larger cages with a cohort of eight animals supplied with nesting and bedding material as well as repeatedly rearranged toys for a total period of six weeks after weaning (Supplementary Fig. S1). In a second cohort, pups were separated daily for 4 h from their mothers from day 1 to 14 after birth. Afterwards, till the age of nine weeks, they were housed in the standard environment like the control group with four animals per standard cage (Supplementary Fig. S1). Body weight for animals in all experimental groups increased significantly over time with no observable difference between WT and BACHD (TG) animals (Supplementary Fig. S2) as shown before^29^. While rats in the MS group had a lower weight relative to the SE groups (Supplementary Fig. S2A), there were no significant body weight differences between the EE and SE groups (Supplementary Fig. S2B). Comparing MS to SE, animals showed an age effect (F_(5,168)_=1190; *p*<0.0001) as well as a treatment effect (F_(1,168)_=27.48; *p*<0.0001), but no genotype differences. Additionally, there were no significant interactions indicating that treatment effects were unspecific for age or genotype. Comparing EE to SE, animals showed an effect of age (F_(5,168)_=996.9; p<0.0001), but not of treatment, genotype, or their interaction.

**Figure 1:**
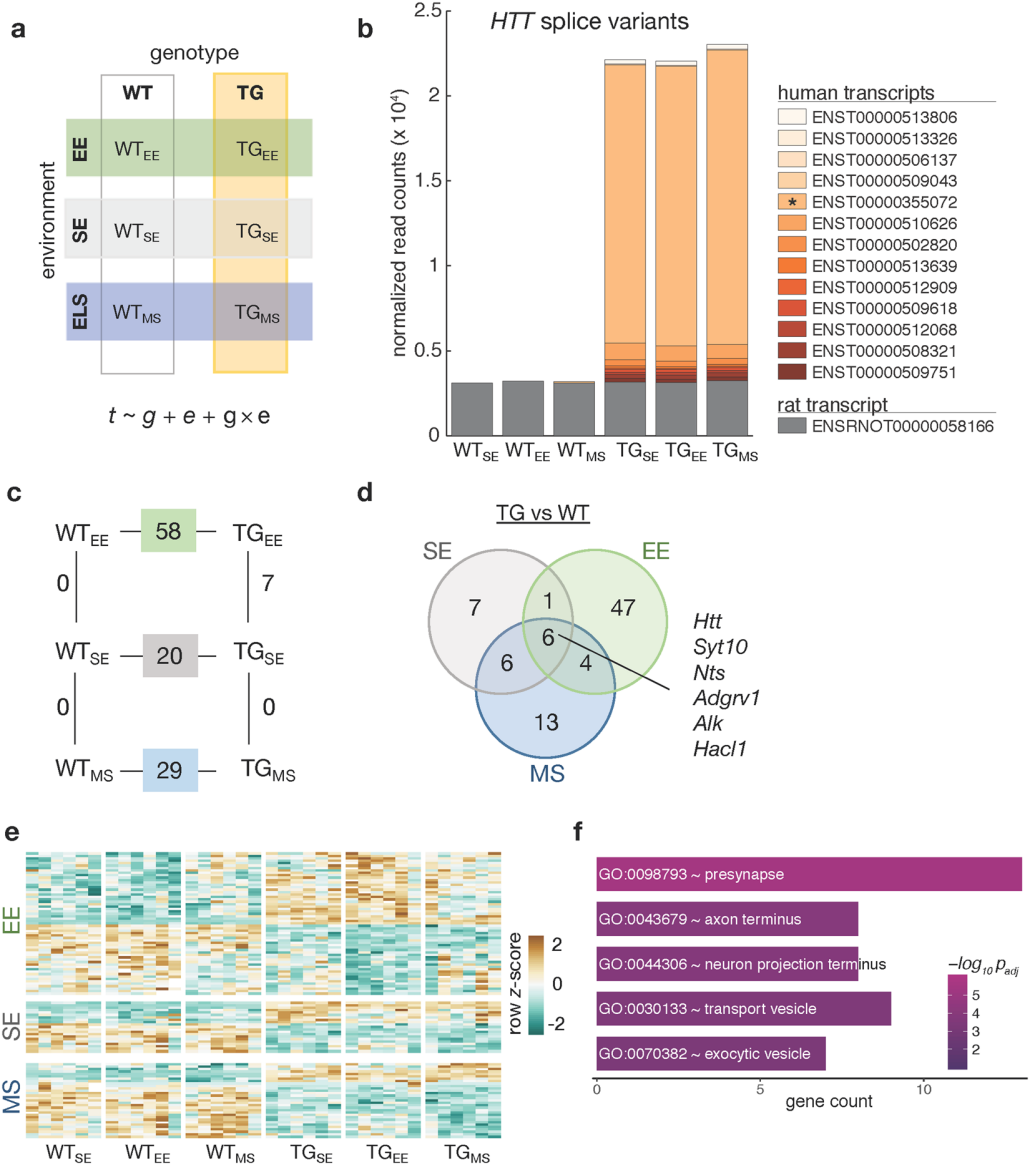
Full-length *mHTT* disturbed the striatal transcriptome. (**a**) Schematic diagram of six experimental groups in a 2 × 3 factorial design based on two genotypes (WT, TG) and three environmental conditions (SE, EE, MS) that was used to reveal effects of environmental enrichment and maternal separation on the striatal transcriptome of 2-month-old WT and BACHD rats. Gene expression (*t*) was modelled as a function of genotype (*g*), environment (*e*), and their interaction (*g* × *e*). (**b**) Composition and expression level of rat and human *HTT* splice variants in the striatum of 2-month-old rats with respect to genotype and environmental condition. (**c**) Diagram showing number of differentially expressed genes between main comparisons in the factorial design. (**d**) Venn diagram showing overlaps of differentially expressed genes between BACHD and WT animals in each of the tested environmental conditions. (**e**) Heatmap of hierarchically clustered *z*-scores across all experimental groups for differentially expressed genes that were identified in comparing TG_SE_/WT_SE_, TG_EE_/WT_EE_, and TG_MS_/WT_MS_. (**f**) Overrepresented Gene Ontology terms of merged differentially expressed genes in TG_SE_/WT_SE_, TG_EE_/WT_EE_, and TG_MS_/WT_MS_.

Using an experimental design that describes all six animal groups with respect to genotype (wildtype, WT; transgenic, TG) and environmental condition (SE, EE, and MS) and modelling gene expression as a function of genotype, environment, and their interaction allowed determining differentially expressed genes across all pair-wise comparisons (Fig. 1a).

Specifically, examining levels of endogenous as well as *mHTT* firstly, we observed no change in the endogenous rat transcript but an intriguing addition of human transcripts in transgenic animals irrespective of the environmental condition (Fig. 1b). The human protein-coding isoform spanning all 67 exons represented the largest fraction. In total, a ~7-fold increase of *HTT* expression was observed, in contrast to the 4.5-fold increase previously reported^29^ which likely results from the more sensitive RNA-seq as well as the alignment against the *mHTT* construct-extended rat genome compared to the previous microarray-based interrogation of the transcriptome.

In addition, overexpression of *mHTT* led to 20 differentially expressed genes (DEGs) in the striatum in the standard environment (TG_SE_/WT_SE_), 58 DEGs under environmental enrichment (TG_EE_/WT_EE_), and 29 DEGs after maternal separation (TG_MS_/WT_MS_) (Fig. 1c). In these three comparisons, six common DEGs were consistently identified irrespective of the environmental condition (Fig. 1d). Besides *HTT*, *Alk* was up- and *Syt10*, *Nts*, *Hacl1*, and *Adgrv1* downregulated (Supplementary Fig. S3). For the latter, we identified a transcript isoform-specific downregulation (Supplementary Fig. S3b).

While the relatively small overlap of only six genes suggested an environment-dependent modulation of the *mHTT*-induced disturbances, most genes showed similar trends of expression changes across the environmental conditions (Fig. 1e). Therefore, merging the DEGs of all three TG/WT comparisons allowed investigating the general transgenic effect on cellular functions resulting in overrepresented Gene Ontology terms including *presynapse*, *axon terminus*, *neuron projection terminus*, *transport vesicle*, and *exocytic vesicle* (Fig. 1f) that were in line with previous findings^6^.

### Early changes in striatal gene expression agree with dysregulation in later stages

**Figure 2:**
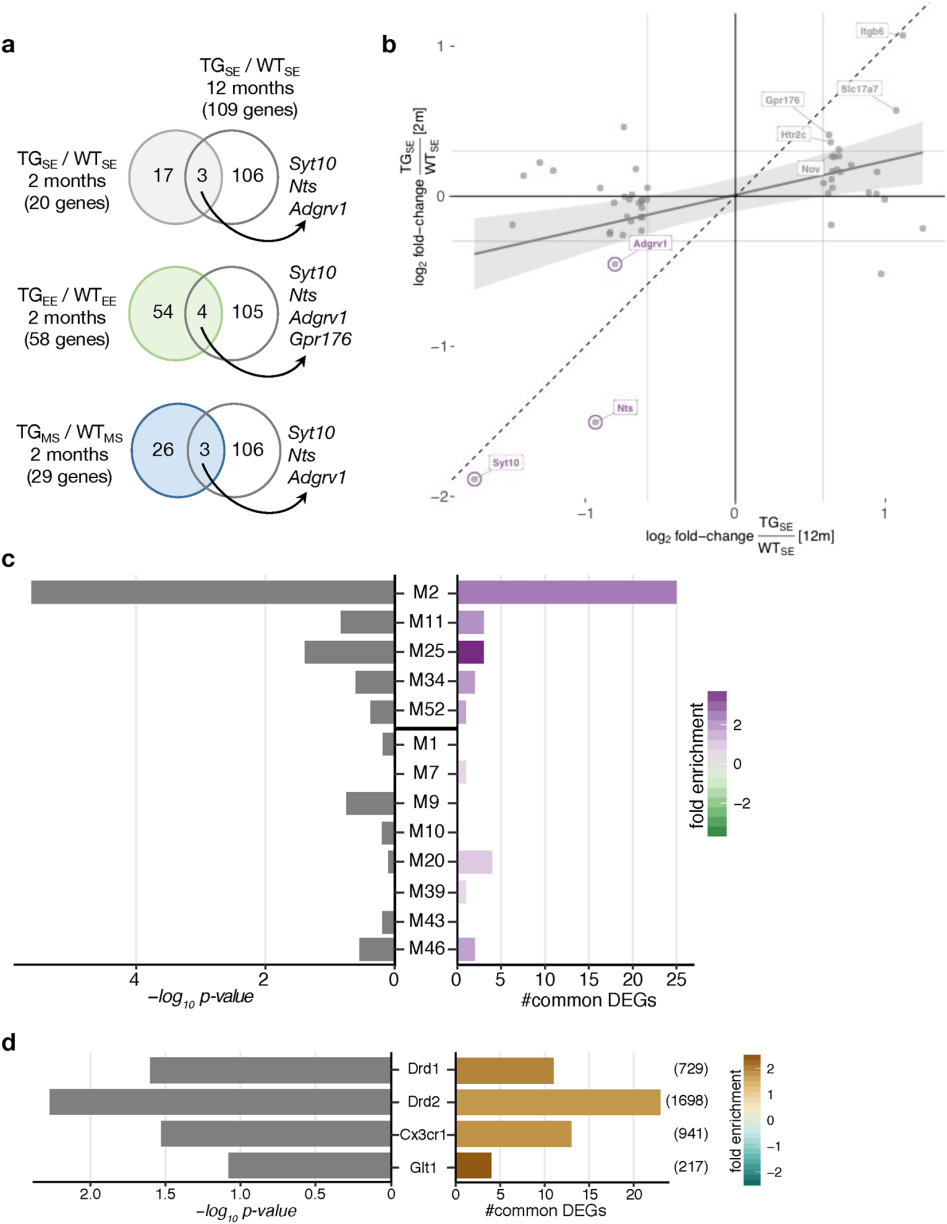
Later-state dysregulation of striatal gene expression induced by *mHTT* surfaced early on. (**a**) Venn diagram comparing differentially expressed genes in 2-month-old BACHD rats housed in one of the three tested environmental conditions with 12-month-old BACHD rats housed in the standard environment^12^. (**b**) Scatter plot of gene expression changes between WT and BACHD rats at 2 months (*x*-axis) and 12 months of age (*y*-axis). Striatal DEGs in 12-month-old BACHD rats^12^ plotted in grey. Striatal DEGs also identified in 2-month-old rats labelled in purple. Linear regression line with standard error shown in grey. Dashed line represents ordinary diagonal. Grey labels indicate DEGs that reached significance in 12-month-old but not in 2-month-old rats. (**c**) Enrichment of DEGs in 2-month-old rats (TG/WT for all three environments) for genes in HD consensus modules^11^. Genes in the upper five modules are downregulated and in the lower eight modules upregulated in a CAG length-dependent manner. Bars show *p*-values of two-sided Fisher’s exact test and number of common genes. (**d**) Enrichment of DEGs in 2-month-old rats (TG/WT for all three environments) for distinct striatal cell types characterized by sorting *Drd1*-, *Drd2*-, *Glt1*-, and *Cx3cr1*-positive striatal cells in mice. Number of cell type-specific genes (p_*adj*_ < 0.1) indicated in brackets on right. Bars show *p*-values of two-sided Fisher’s exact test and number of common genes.

To relate these DEGs, identified at an early age, to later changes in the context of HD, we compared them with striatal DEGs of 12-month-old BACHD rats^12^. When comparing gene expression changes under SE conditions between 2 and 12 months of age for the 109 DEGs identified at the later time point, similarities of dysregulation were observed (Fig. 2b). The strongest correlation was found for *Syt10*, *Nts*, and *Adgrv1* that showed differential expression across all three environmental treatments (Fig. 2a), suggesting these genes to represent characteristic markers for early *mHTT* disease unfolding irrespective of environmental condition.

To further explore these age-dependent effects, we integrated our data with available data from striatal consensus modules based on *HTT*-CAG length and age-dependent gene co-expression networks^11^. The most significant enrichment of DEGs in these modules was observed for module M2, M11, and M25, which contain genes that become increasingly downregulated with CAG length and age (Fig. 2c). Module M2 contains genes involved in *cAMP signalling*, while modules M11 and M25 are enriched for glia-related genes^11^.

The link to glia-related disturbances was further supported by comparing DEGs in BACHD rats to genes attributed to distinct striatal cell types in mouse. Differential expression in BACHD rats correlated significantly with the expression of their orthologous genes in *Glt1*-, and *Cx3cr1*-positive striatal cells in mice (Fig. 2d). In these cell type-specific comparisons, the most significant enrichment was found between BACHD DEGs and genes attributed to striatal medium spiny neurons sorted by *Drd2* (Fig. 2d), in line with the critical role of this cell type in the context of *mHTT *^37^.

### Genotype-dependent activation of genes in response to environmental enrichment

**Figure 3:**
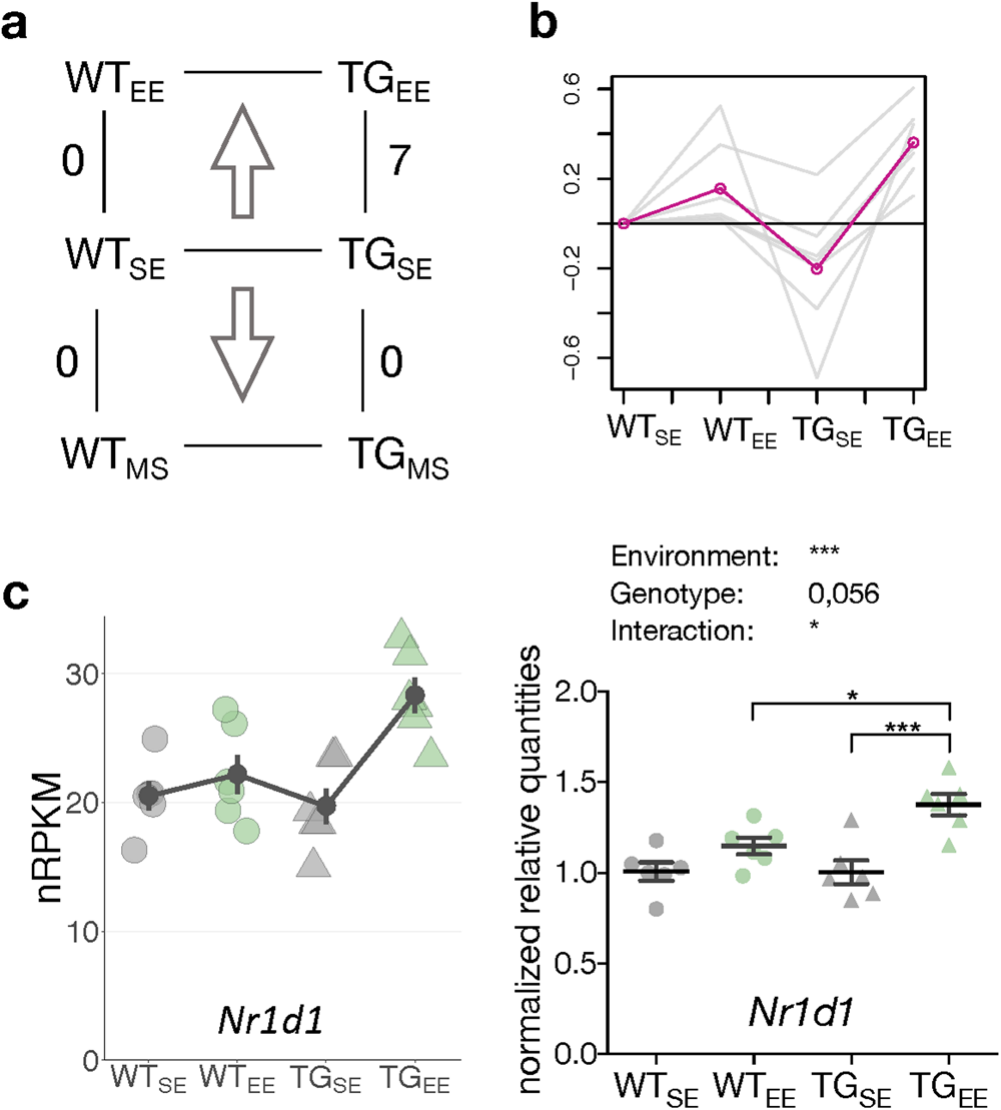
Environmental enrichment activated genes specifically in transgenic animals. (**a**) DEGs comparing WT and TG rats in EE and MS. (**b**) Gene expression profiles of seven DEGs in TG_EE_/TG_SE_ relative to WT_SE_ across four experimental groups. Mean profile in pink. (**c**) Left panel shows expression level for *Nr1d1* plotted as individual data points with mean ± SEM. Right panel shows quantitative PCR data for *Nr1d1* as normalized quantities relative to WT_SE_ (n = 6 mice per group) plotted as individual data points with mean ± SEM. Two-way ANOVA was performed followed by Tukey’s multiple comparisons test. *p < 0.05, ***p < 0.001.

Having integrated early striatal gene expression changes in BACHD rats with age-dependent aspects of HD, we next explored the impact of environmental changes in wildtype and transgenic rats. Intriguingly, neither MS nor EE led to any differentially expressed genes in the striatum of WT rats (Fig. 3a). Genes such as *Oprk1*, *Nrxn1*, and *Nfia*^38,39^ that are known to be responsive to early life stress showed trends of differential expression (Supplementary Fig. S4). Other markers such as *Bdnf* and *Il1b* showed very low expression in the striatum making conclusive results difficult to derive (Supplementary Fig S4). In TG animals, no DEGs were identified upon MS, however, seven genes (*Ankrd13b*, *Clcf1*, *Dnajc30*, *LOC681*2*8*2, *Nr1d1*, *Slc22a3*, and *Syndig1l*) were significantly upregulated upon EE (Fig. 3a,b). We confirmed this genotype-specific response with qPCR on *Nr1d1* as a representative of their activation pattern (Fig. 3c).

### Environmental enrichment modulated several genes against their later-stage trend of dysregulation

**Figure 4:**
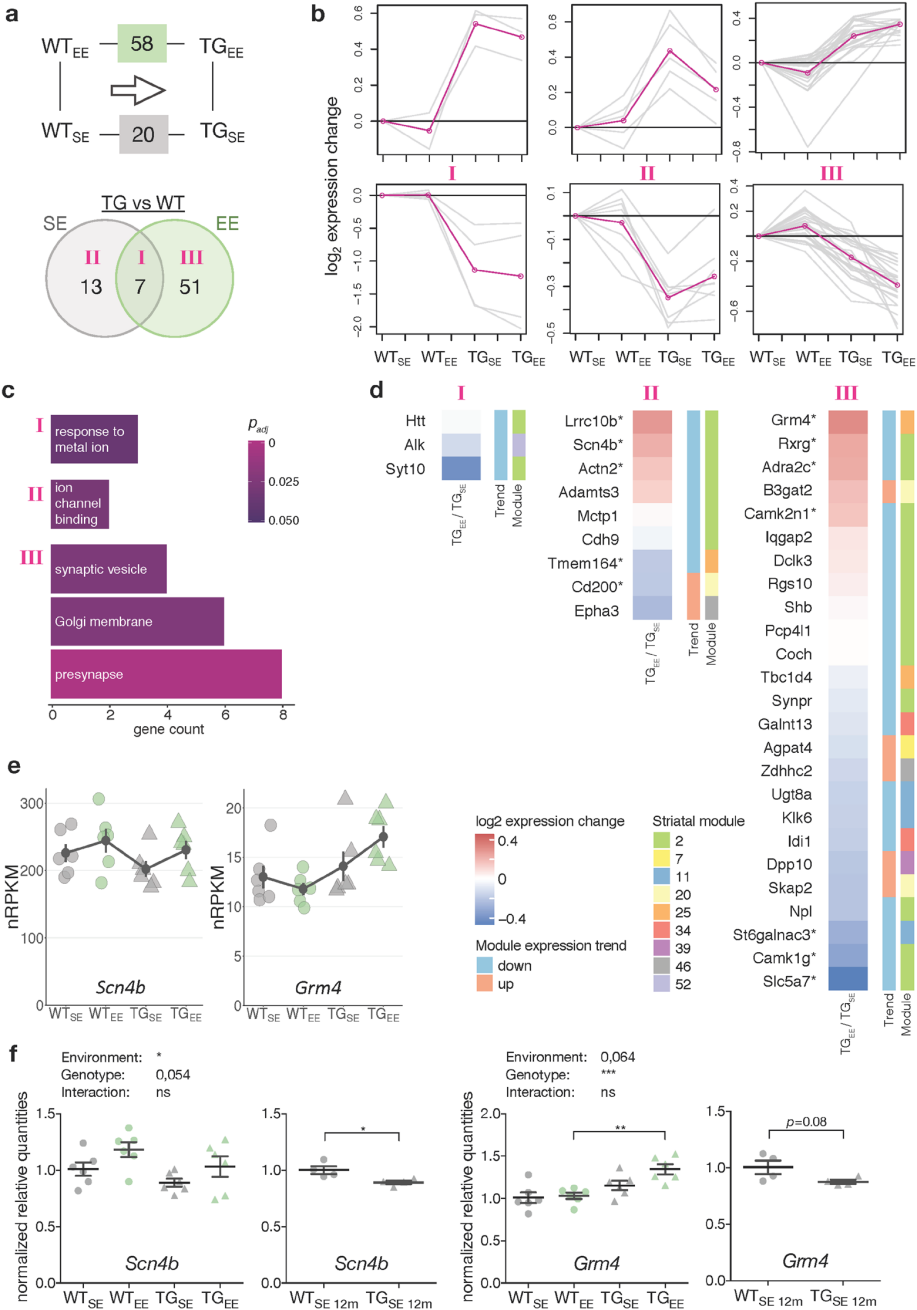
Environmental enrichment modulated striatal expression of disturbed genes. (**a**) Schematic and Venn diagram comparing number of DEGs between TG_SE_/WT_SE_ and TG_EE_/WT_EE_. Roman numbers indicate sets analysed individually in (**b**). (**b**) Cluster analysis of the subsets of DEGs in (**a**) using *k*-means on gene expression ratios relative to WT_SE_ across four experimental groups. Grey lines indicate expression changes per gene, pink lines cluster centroids. (**c**) Gene count and significance of overrepresented Gene Ontology terms for subsets of DEGs in (**a**). (**d**) Subsets of DEGs in (**a**) plotted with respect to their expression change between TG_EE_/TG_SE_, consensus module association and trend^11^. DEGs with no module information omitted. Rows sorted by mean expression change. Asterisks indicate DEGs with *p*-value < 0.05 when comparing TG_EE_/TG_SE_. (**e**) Expression levels for *Scn4b* and *Grm4* plotted as individual data points with mean ± SEM. (**f**) Quantitative PCR data for *Scn4b* and *Grm4* shown as normalized quantities relative to WT_SE_ (n = 6 and n = 4 rats per group in 2-month-old and 12-month-old cohorts, respectively) plotted as individual data points with mean ± SEM. Significance determined by two-way ANOVA followed by Tukey’s multiple comparisons test for the 2-month-old cohort, and unpaired two-tailed *t*-test for the 12-month-old cohort. *p < 0.05, **p < 0.01, ***p < 0.001.

After having explored the transgenic and environmental impact on the striatal transcriptome individually, we studied their combined influence in order to assess environment-dependent modulation of gene expression changes in the context of *mHTT*. Focusing on the 58 DEGs in TG_EE_/WT_EE_ and comparing them to the 20 DEGs found in TG_SE_/WT_SE_, seven overlapped (class I), while 13 were exclusively identified in SE (class II), and 51 DEGs were exclusively found in EE (class III) (Fig. 4a). To better understand the characteristics of the genes in these classes, their expression profiles were clustered (Fig. 4b). Class I was enriched for the GO term *response to metal ion*, and class II for *ion channel binding* (Fig. 4b,c). The class II expression profile reflects the intuitive amelioration, yet no complete prevention, of disturbed gene expression through the EE. Class III contained genes overrepresented for the GO terms *synaptic vesicle*, *Golgi membrane*, and *presynapse* (Fig. 4b,c) and stronger differential expression in EE than in SE, counter-intuitive to a protective influence at first glance.

To interpret these modulations through the EE, we compared the DEGs in these classes with their associated consensus module characteristics^11^. For most DEGs in class II the modulating effect of the EE between TG_EE_/TG_SE_ was indeed against their associated module trend, further supporting the intuitive ameliorating effect (Fig. 4d). Intriguingly, the modulation of expression changes through the EE in class III, in particular on upregulated genes, was against the predicted changes according to the consensus modules, too (Fig. 4d). This result resolves the contradiction of alleged aggravated disturbances and renders the EE-induced modulations into ameliorations against the age trend.

To validate these results, gene expression changes were measured for selected candidates in 2- and 12-month-old rats using quantitative PCR. In line with the computationally predicted expression changes, *Scn4b* in class II and *Grm4* in class III were downregulated at 12 months (Fig. 4e,f), and showed less downregulation at 2 months of age in EE conditions (Fig. 4e,f).

Maternal separation aggravated expression changes of some genes towards their later-stage level of dysregulation.

**Figure 5:**
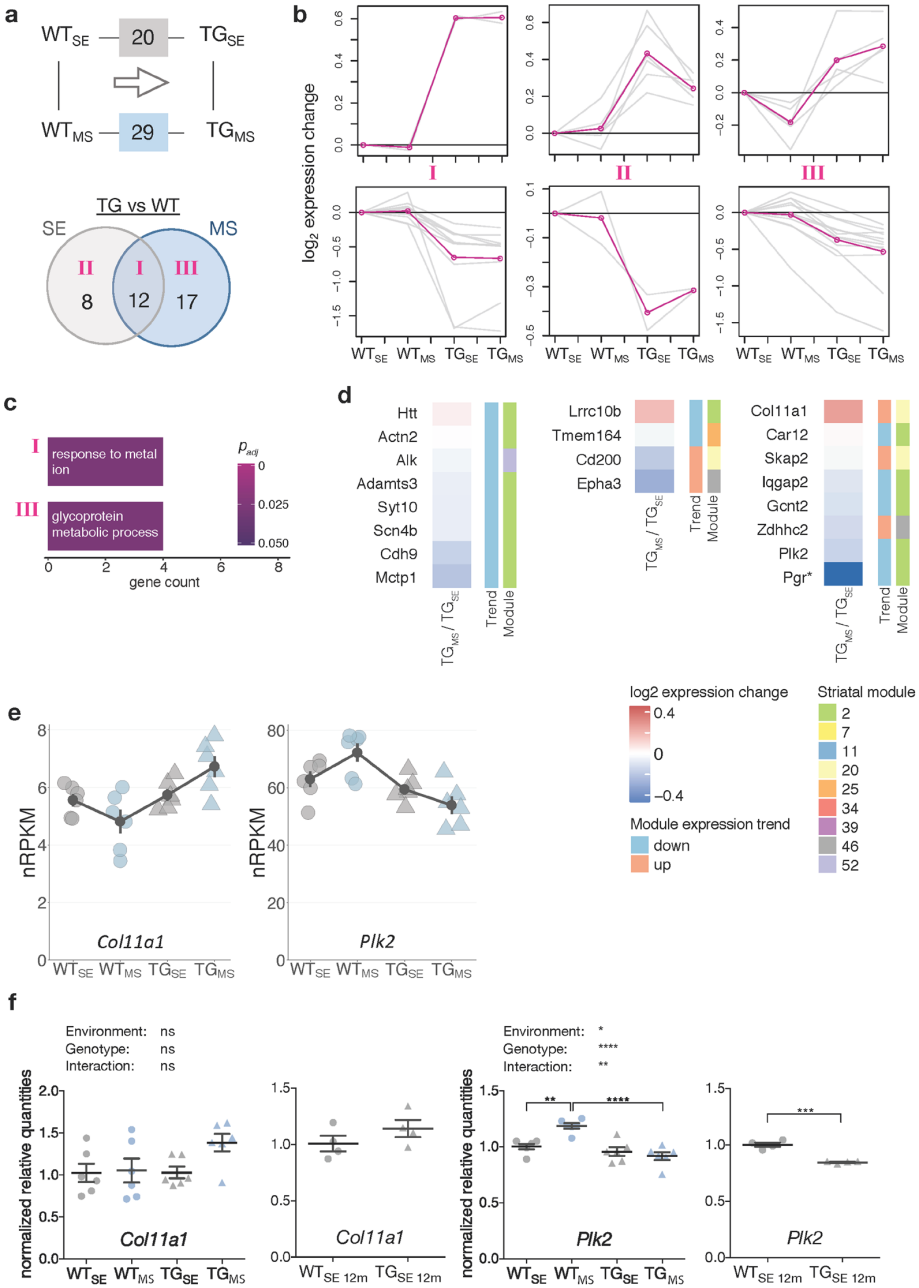
Maternal separation modulated striatal expression of disturbed genes. (**a**) Schematic and Venn diagram comparing number of DEGs between TG_SE_/WT_SE_ and TG_MS_/WT_MS_. Roman numbers indicate subsets analysed individually in (**b**). (**b**) Cluster analysis of the DEG subsets in (**a**) using *k*-means on gene expression ratios relative to WT_SE_ across four experimental groups. Grey lines indicate expression changes per gene, pink lines cluster centroids. (**c**) Gene count and significance of overrepresented Gene Ontology terms for subsets of DEGs in (**a**). (**d**) Subsets of DEGs from (**a**) plotted with respect to their expression change between TG_MS_/TG_SE_, module association, and trend^11^. DEGs with no module information omitted. Rows sorted by mean expression change. Asterisks indicate DEGs with *p*-value < 0.05 when comparing TG_MS_/TG_SE_. (**e**) Expression levels of *Col11a1* and *Plk2* plotted as individual data points with mean ± SEM. (**f**) Quantitative PCR data for *Col11a1* and *Plk2* shown as normalized quantities relative to WT_SE_ (n = 6 and n = 4 rats per group in 2-month-old and 12-month-old cohort, respectively) plotted as individual data points with mean ± SEM. Significance determined by two-way ANOVA followed by Tukey’s multiple comparisons test for the 2-month-old cohort, and unpaired two-tailed *t*-test for the 12-month-old cohort. *p < 0.05, **p < 0.01, ***p < 0.001, ****p < 0.0001.

After exploring EE-induced modulations, we next focused on the impact of MS on *mHTT*-induced striatal gene expression disturbances. Compared to 20 DEGs in TG_SE_/WT_SE_, 29 DEGs were identified in TG_MS_/WT_MS_ (Fig. 5a). Similar to the approach described for the EE, we grouped the three subsets of genes (class I, II, III) based on overlaps between both comparisons (Fig. 5b). Class I was enriched for the GO term *response to metal ion* (Fig. 5c). Class II, containing genes whose transgene-induced disturbance in SE was ameliorated after MS, was not enriched for any GO term. Interestingly, some of these genes responded similar under EE and MS conditions (Supplementary Fig. S5). Class III contained DEGs with stronger gene expression changes after MS, which were enriched for the GO term *glycoprotein metabolic process* (Fig. 5c).

Computationally associating genes in these three classes with consensus module information, an ameliorating effect for most class II genes against the module trend was apparent similar to effect observed in EE (Fig. 5d, Supplementary Fig. S5). In contrast, for several genes in class III the stronger expression changes after MS pointed in the same direction as the predicted changes according to the consensus modules, thereby representing a reinforcement of the transgenic disturbance expected with age by MS (Fig. 5d). These computational associations were confirmed for *Col11a1* and *Plk2* using quantitative PCR. *Col11a1* indeed showed upregulated levels with age and after MS already in young animals (Fig. 5e,f). *Plk2* was downregulated with age similar to its response to MS in young animals (Fig. 5e,f).

### Disease aspects and biofunctions associated with *mHTT* suggest distinct responsiveness to enriched environment and maternal separation

**Figure 6:**
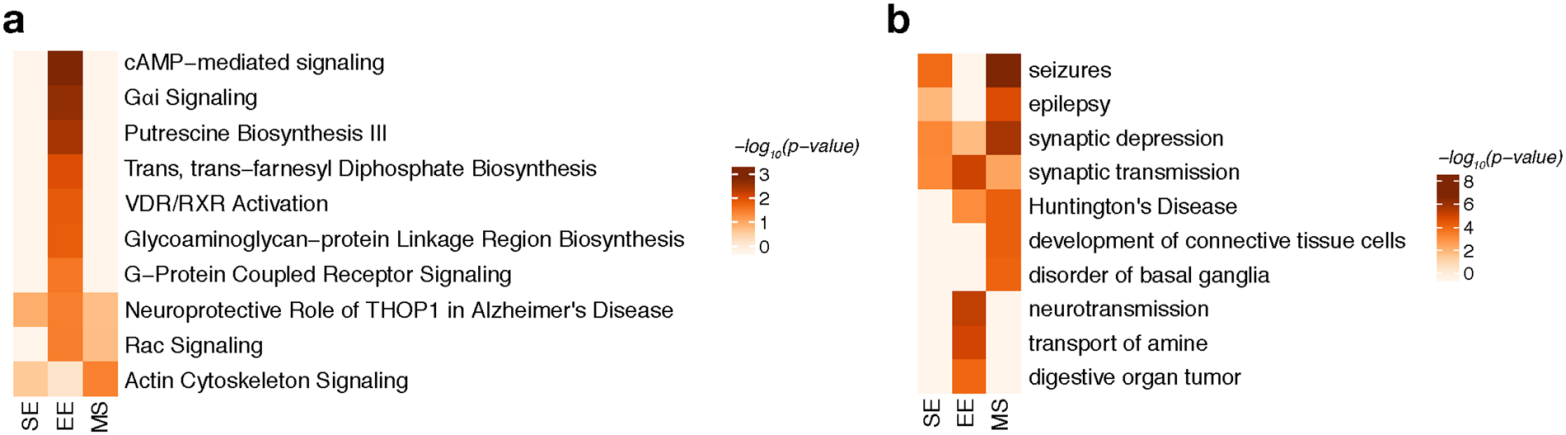
Shared and distinct characteristics in pathways and disease aspects for BACHD rats after environmental enrichment and maternal separation. (**a**) Canonical pathway analysis identified significantly enriched cellular pathways among DEGs derived in the tested environmental conditions for BACHD rats. Significance values colour-coded and hierarchically clustered representing likeliness of pathway participation. (**b**) Disease aspects and biological functions predicted to be affected based on DEGs derived in the tested environmental conditions. Significance values colour-coded and hierarchically clustered representing likeliness of downstream effects on disease aspects.

For several genes, EE as well as MS modulated the *mHTT*-induced gene expression disturbances (Figs 4 and 5), while other genes remained altered irrespective of the environment. Using pathway analysis tools, we investigated this environmental modulation further. Few pathways were detected to varying significance across all environmental conditions such as *actin cytoskeleton signalling* and *Rac signalling* (Fig. 6a). Additional pathways originated mostly from the comparison in EE including *cAMP signalling*, *VDR/RXR activation*, and *G-protein coupled receptor signalling* (Fig. 6a).

Exploring these pathways with respect to disease aspects in TG animals, we found *seizures*, *epilepsy*, and *synaptic depression* to be more significant for MS DEGs compared to the SE or EE groups (Fig. 6b). In contrast, *synaptic transmission* and *neurotransmission* were more significant in EE, while they seemed less involved in SE or MS (Fig. 6b), agreeing with previously described protective effects of environmental enrichment^20^.

### Validation of specific candidate genes on protein level

To quantify protein levels of *NR1D1*, *mGluR4* (*GRM4*), and *SCN4B* which showed environment-dependent gene expression changes Western blot assays were performed (Supplementary Fig. S6). Additionally, protein levels of *mHTT* were quantified even though *mHTT* gene expression was not significantly affected by any environmental condition (Supplementary Fig. S6).

Although not significant, *NR1D1* protein levels showed similar tendencies to those observed for the respective mRNA with increased expression in TG_EE_ compared to TG_SE_ and WT_EE_ (Supplementary Fig. S6a). Opposite to the gene expression results, protein levels of *mGluR4* (*GRM4*) were significantly lower in TG than in WT rats irrespective of the environmental condition (Supplementary Fig. S6a). Statistical analyses showed a genotype but no environment effect for *mGluR4* protein levels. Moreover, no significant environment or genotype effects were detected for *SCN4B* (Supplementary Fig. S6a).

Intriguingly, protein levels of *mHTT* were reduced in TG_EE_ relative to TG_SE_ (Supplementary Fig. S6a) while they were comparable between TG_MS_ and TG_SE_ (Supplementary Fig. S6b) which suggests a specific, potentially protective, effect of environmental enrichment on *mHTT* protein levels.

## Discussion

Taken together, the overall aim of this study was to assess environmental effects on striatal gene expression in young BACHD rats. Our results show that *mHTT*-induced gene expression changes (i) correlated between younger and older animals, (ii) were dependent on the environment, and (iii) were partially modulated against and with their trend of dysregulation at later ages through environmental enrichment and maternal separation, respectively.

Specifically, we observed a dominant effect of *mHTT* on striatal gene expression changes across the tested environmental conditions. Combining differentially expressed genes from these comparisons were enriched for Gene Ontology terms that agree with previously reported disturbances of *mHTT* on cellular vesicle transport and secretion as well as synaptic transmission and vesicle exocytosis^40–42^.

Further, we found changes in gene expression induced by *mHTT* at young age to agree with disturbances observed in later stages of the disease in this model. Differentially expressed genes found in young and older BACHD rats suggest that these genes may serve as characteristic markers of disease unfolding. Candidates in this overlap such as *Syt10*, a vesicular Ca^2+^-sensor with neuroprotective potential^43^, and *Nts* a modulator of dopaminergic transmission known to be dysregulated in HD^37^, were downregulated and point at aspects of the disease that are disturbed early on.

Support for the idea that disease aspects are already detectable at this young age can be derived from the significant overlap of DEGs with some of the most informative HD consensus modules from co-expression meta-analyses^11^. Furthermore, these disturbances seem characteristic for the disease as consensus module data from HD mouse models largely agree with gene expression changes in BACHD rats.

Also on cell type level, our findings support the critical role of D2 neurons for the developing pathology in this disease model, agreeing with this cell type’s high vulnerability in HD^44^, and strengthen the emerging concept that also glial-cell populations are affected. Glia impairments have been described in R6/2 mice^45^ and overexpression of *mHTT* in glia cells has been shown to worsen the HD phenotype in N171–82Q mice^46^, suggesting a role of glia cells in HD pathology to be further followed up functionally in BACHD rats.

Both tested environmental conditions partially modulated the *mHTT*-induced disturbances. Environmental enrichment affected gene expression changes against the consensus module trend suggesting a preventive effect in disease progression. Intriguingly, this prevention exhibited not only an ameliorating effect on some disturbed genes, but also led to stronger expression changes for other genes. Despite being counter-intuitive, the latter modulation type seems to represent a response of the system to the EE in order to prevent disturbances appearing later in time. Among the genes perceiving an amelioration of their disturbed expression was *Scn4b*, encoding the 4β subunit of a voltage dependent sodium channel implicated in neurite growth^47^. Its downregulation in young and old BACHD rats agrees with previous reports on impaired *Snc4b* expression in R6/2 mice and HD patients already at a pre-symptomatic stage^47^ suggesting a critical role of this gene in the degenerative processes in HD. *Grm4*, on the other hand, was among the genes responding to the EE by stronger upregulation. It encodes for the glutamate metabotropic receptor 4, and is a hub gene in consensus module M25, whose genes are downregulated and overrepresented for *glutamate receptor signalling*^11^. In addition, *Grm5*, encoding a receptor of the same protein family, has been linked to HD-like impairments in Hdh(Q111/Q111) mice^48^ and implicated in hippocampal effects of environmental enrichment^49^. The observed upregulation of *Grm4* in TG_EE_ animals might, thus, be part of known protective responses of EE^23^, and are in line with previously reported findings of metabotropic receptors in neuroprotection^50^.

In contrast to the EE, maternal separation aggravated disturbed gene expression of some genes towards their later stage trend of dysregulation in young BACHD rats. *Plk2*, for example, was among the genes that were further downregulated by MS in the context of *mHTT*. Activation of *Plk2* has been shown in response to early insults such as prenatal stress and neonatal seizures suggesting a role in hippocampal synaptic downscaling and supporting the idea that *Plk2* might be protective^51^. In keeping, *Plk2* was increased in WT animals subjected to maternal separation. BACHD rats, in contrast, showed decreased expression that was further decreased after MS, in line with a general aggravation of HD markers upon stress experiences^27,28,52^. This aggravating effect further suggests greater sensitivity of BACHD rats to stressful experiences agreeing with hyperactivity of the hypothalamic-pituitary-adrenal axis in HD patients^53^.

Phenotypically, we observed lower body weights for rats in the MS group as it has been previously reported as a consequence of maternal separation^54^ as well as in response to stressors in adult rats^55,56^ suggesting body weight loss to reflect stress here, too. We did not assess direct physiological stress responses (e.g. body temperature or stress hormones) to limit manipulations of the animals. Measuring stress hormone levels when rats were sacrificed would have potentially allowed assessing basal stress activity^57,58^. Yet, they have also been found comparable to control levels^58–61^.

Behavioural tests may also allow assessing MS effects and are essential to link gene expression changes to phenotypic consequences in the context of environmental factors. Here, we however sought to minimize disturbances of the animals through behavioural testing and handling as they may confound gene expression changes^62^. Moreover, only part of the behavioural phenotypes described for BACHD rats have been examined at such early age^29–32,63,64^. Follow-up studies that consider later time points will enable investigations whether behavioural and physiological characteristics in the BACHD rat model depend on environmental factors. In order to determine preventive and aggravating aspects of environmental factors in the unfolding of the disease, rats would have to be kept in standardized environments for longer periods. Having data from such later time points will then allow resolving disease progression and environmental impact in greater detail and facilitate analyses of their interplay with respect to temporal delays or accelerations on HD pathology. To additionally investigate whether there are any EE-driven rehabilitative effects or reversals of dysfunctions animals could first be housed in SE or MS until developing initial signs of pathology and then migrating them to the EE.

On a molecular level, typical markers such *Bdnf* and *Il1b* showed very low expression in striatal tissue and did not allow deriving statistically significant results. Other genes including *Oprk1*, *Nrxn1*, and *Nfia*^38,39^ that are known to be responsive to stress showed trends of changed expression in WT rats after maternal separation. However, no significantly expressed genes were identified comparing TG_MS_/TG_SE_ or WT_MS_/WT_SE_, possibly related to general timings in the developmental gene regulatory program as many essential genes for striatal function and relevant in the context of HD may not yet be fully expressed during the first two postnatal weeks^65^. Additionally, housing animals in groups of four under standard conditions after the stress paradigm may have masked early stress effects as enriched conditions can even reverse them^36^.

Agreeing with previous reports on few differentially expressed genes in the striatum upon environmental enrichments in the context of *mHTT*^9^, we identified seven distinctively activated genes specifically in BACHD rats. Among them was *Nr1d1* encoding a transcription factor of the nuclear receptor subfamily 1 (Rev-erbα) that is involved in regulating circadian rhythm and metabolic processes^66^. Given the metabolic deficits of young BACHD rats including obesity and altered levels of energy metabolism factor^64^, upregulation of *Nr1d1* in BACHD rats upon EE may hint at relevant processes as to how environmental enrichment affects metabolic phenotypes. Collecting metabolic parameters known to be altered in BACHD rats^29,64^ will help to follow up this gene-environment interaction.

To reveal whether gene expression changes were present at protein level too, we measured protein levels of *NR1D1*, *mGluR4* (*GRM4*), and *SCN4B* for which environment-dependent gene expression changes were shown and partially confirmed mRNA results on protein level.

Such discrepancies between mRNA and protein levels have been observed for a variety of proteins and may originate from both biological as well as methodological aspects^67^. A poor correlation between mRNA and protein levels in our samples may be explained by post-translational mechanisms regulating protein turnover and abundance^67^. These mechanisms may explain the opposite pattern of changes for *Grm4* mRNA and its protein. Although not in line with mRNA changes, this effect is interesting given the neuroprotective function of *mGluR4* and other group III metabotropic glutamate receptors^68,69^. Therefore, *mGlur4* downregulation may represent a potential mechanism mediating neuropathological alterations in BACHD rats.

Alternative reasons for the weak correlation between mRNA and protein changes could result from technical limitations. The lower sensitivity of Western blot analysis relative to RNA-seq and qPCR, and the high variability among Western blot replicates may have limited the detection of small changes. This could in part explain the lack of significant EE effects on *NR1D1* and *SCN4B* protein levels, despite the similar pattern of mRNA and protein levels.

By affecting post-translational events and other regulatory mechanisms, the environment might also affect protein levels without altering mRNA expression. Accordingly, EE downregulated *mHTT* protein without altering its mRNA levels. The underlying mechanisms of EE on *mHTT* in BACHD rats will be interesting to follow up considering the central role of *mHTT*.

In summary, results of this study show disturbances on striatal gene expression by *mHTT* in BACHD rats that are partially dependent on environmental condition early in life. Bivalent modulations of disturbed genes in response to environmental enrichment or maternal separation may, thus, contribute to explain their opposing phenotypic effects in HD and related neurodegenerative diseases. In order to better connect these disease aspects with disturbances on gene expression level, it will be critical to also interrogate protein abundancies, physiological parameters, and behavioural phenotypes.

## Materials and Methods

### Animals, study design and environmental treatments

Experiments were performed in BACHD rats and WT littermates. The BACHD rat model was generated using a BAC containing the whole human *HTT* locus with 20 kb upstream and 50 kb downstream flanking genomic sequences including regulatory elements^29^. WT *HTT* exon 1 was replaced by *mHTT* exon 1 carrying 97 mixed CAA/CAG repeats flanked by two LoxP sites^29^.

All animals were bred on a Sprague Dawley background by pairing heterozygous BACHD males with wildtype females (Charles River, Germany). At the day of birth, indicated as postnatal day (PD) 0, all litters were culled to eight pups ensuring equal numbers of females and males similar to previous maternal separation experiments^58,59,70^.

In order to avoid separating littermate pups during maternal separation and to keep comparable conditions for all experimental groups, a litter, including both male and female pups, was randomly allocated to one of three environmental paradigms: standard environment (SE), maternal separation (MS), and enriched environment (EE) (Supplementary Fig. S1). Each environmental paradigm (n = 32) included four groups of WT males, WT females, TG males, TG females, (n = 8 per group), totalling to 96 animals. To avoid variability of the data based on hormonal influences in cycling female rats only male rats were used for RNA-seq which was performed on tissue from six rats that were randomly chosen from each of the six environmental groups (n = 36).

The maternal separation protocol was adapted from previous studies^58,61,71^. In brief, from PD 1 to 14, pups were separated from their mother for 4 h daily starting at the beginning of the light phase. Separated pups were placed in glass containers leaned with paper and nesting material and transported to a different room to prevent communication between pups and dams. In order to maintain comparable temperatures (32–33 °C) between pup environment in the home cage and the nest during separation, pups were kept in containers placed in a warm water bath.

At PD 16, rats were genotyped following established protocols^29^. At PD 21, rats were weaned. Rats of the EE group were housed in groups of eight animals in larger cages (1222 × 580 × 443 mm) enriched with bedding, nesting material, toys, and objects of different material, colour, and shape for the entire duration of the experiment. Novel objects were introduced every 3–4 days. Rats in other experimental groups were housed in groups of four in standard type IV cages (598 × 380 × 200 mm) equipped with bedding and nesting material. Control animals were left undisturbed for the entire experiment except for cage cleaning and body weight measurements that were collected weekly from weaning throughout the experiment in all experimental groups. All rats were housed with same-gender littermates of mixed genotype and maintained in a room with constant temperature (22 ± 1 °C), humidity (55 ± 10%), and a regular 12 h light/dark cycle (lights on/off at 7 AM/7 PM). Food and water were provided *ad libitum*.

At two months of age (PD 59–60), animals were sacrificed by live decapitation before extracting their brains for dissection. Brain samples were frozen in liquid nitrogen and stored at –80 °C.

RNA from eight additional male rats of the same line (4 WT and 4 BACHD) was used to measure gene expression of selected candidate genes at 12 months of age. These rats were bred in a separate cohort and left undisturbed except for cage cleaning until being sacrificed. Their housing and room conditions were the same as for SE groups except for additional tissue paper in the cage. Protocols applied for live decapitation as well as brain collection, dissection, and storage were as described above. This extra cohort of animals included only WT and TG rats housed in SE. This assay does not represent a second time point in the experiment and was solely used to compare changes at young and later age.

All procedures strictly adhered international standards for the care and use of laboratory animals and were approved by the local Animal Welfare and Ethics committee of the Country Commission Tübingen, Germany (TVA HG 2/15 and TVA HG 6/13).

### Experimental procedures

#### RNA isolation

Total RNA and DNA were simultaneously extracted from striatal samples of 2-month-old rats using the *AllPrep DNA/RNA/miRNA Universal Kit* (Qiagen) following the manufacturer’s protocol. Total RNA was extracted from striatal samples of 12-month-old rats with *RNeasy Mini Kit* (Qiagen) according to the manufacturer’s protocol.

#### RNA sequencing

The polyadenylated fraction of RNA isolated from striatal tissue of 2-month-old rats (n = 6 animals in each of the 6 experimental groups) was used for RNA-seq. Quality was assessed with an *Agilent 2100 Bioanalyzer*. Samples with high RNA integrity number (RIN >8) were selected for library construction. Using the *TruSeq Stranded RNA Sample Prep Kit* (Illumina) and 100 ng of total RNA for each sequencing library, poly(A) selected paired-end sequencing libraries (136 bp read length) were generated according to the manufacturer’s instructions. All libraries were sequenced on an *Illumina HiSeq 2500* platform at a depth of ~15–25 million reads each. Library preparation and sequencing procedures were performed by the same individual, and a design aimed to minimize technical batch effects was chosen. Fastq files and raw counts are available through GEO (GSE107259).

#### Reverse transcription-quantitative PCR (RT-qPCR)

RNA-seq results were validated using RT-qPCR with primers specific for *Nr1d1*, *Snc4b*, *Grm4*, *Col11a1*, and *Plk2* genes (see Supplementary Table S1 for primers sequences). 100 ng of total RNA was used for the reverse transcription reaction (*QuantiTect Reverse Transcription* kit, Qiagen) following the manufacturer’s instructions. The resulting cDNA was diluted (1:20) and 2 µl were used for qPCR assays, mixed with primers (0.25 µM) and *SYBR green* master mix (Qiagen). Expression was calculated relative to the mean of the WT_SE_ group according to the Pfaffl model^72^ after normalization to the geometric mean relative expression of three reference genes (*Pgk1*, *Gapdh*, and *Eif4a2* for 2-month-old rats. *Pgk1*, *Gapdh*, and *Ywhaz* for 12-month-old rats). Expression stabilities of the reference genes were assessed using *Normfinder*^73^ and *Genorm*^74^.

#### Protein extraction and Western blotting

Homogenates of rat striata were obtained by homogenising brain hemispheres in RIPA buffer (50 mM Tris pH 7.5, 150 mM NaCl, 0.1% SDS, 0.5% sodium deoxycholate and 1% Triton X-100, containing cOmplete® protease inhibitor cocktail and PhosSTOP™ phosphatase inhibitors (both Roche)) using an ULTRA-TURRAX® disperser (VWR). Samples were ultrasonicated for 3 s using a SONOPULS ultrasonic homogenizer (Bandelin) at 10% power. Homogenates were incubated for 30 min on ice, mixed with glycerol to a final concentration of 10% and stored at −80 °C for further analysis. Protein concentrations of RIPA homogenates were measured spectrophotometrically using Bradford reagent (Bio-Rad Laboratories).

Protein blotting was performed according to standard procedures. Briefly, 25 µg of total protein were mixed with 4  ×  LDS sample buffer (1 M Tris Base pH 8.5, 2 mM EDTA, 8% LDS, 40% glycerol, and 0.025% phenol red) in a 3:1 ratio and supplemented with 100 mM dithiothreitol. After heat-denaturing for 10 min at 70 °C, protein samples were electrophoretically separated using 4–12% Bolt^®^ Bis-Tris gradient gels, 10% Bis-Tris gels, and 7% NuPAGE^®^ Tris-Acetate gels (both Thermo Fisher Scientific) with respective electrophoresis buffers, MES buffer (50 mM MES, 50 mM Tris Base pH 7.3, 0.1% SDS, 1 mM EDTA), MOPS buffer (50 mM MOPS, 50 mM Tris Base pH 7.7, 0.1% SDS, 1 mM EDTA) or Tris-Acetate running buffer (50 mM Tricine, 50 mM Tris Base pH 8.25, 0.1% SDS). Proteins were transferred on Amersham™ Protran™ Premium 0.2 µm nitrocellulose or Amersham™ Hybond™ P 0.2 polyvinyliden fluoride membranes (both GE Healthcare) using Bicine/Bis-Tris transfer buffer (25 mM Bicine, 25 mM Bis-Tris pH 7.2, 1 mM EDTA, 15% methanol) and a TE22 Transfer Tank (Hoefer) at 250 mA for 1.5–2 h.

After transfer, membranes were blocked for 1 h with 5% skim milk powder (Merck) in TBS (Tris-buffered saline) at room temperature, and probed overnight at 4 °C with the following primary antibodies diluted in TBS-T (TBS with 0.1% Tween 20): rabbit anti-huntingtin (1:1000; clone D7F7, #5656, Cell Signaling), rabbit anti-metabotropic glutamate receptor 4 (1:250; ab53088, Abcam), rabbit anti-NR1D1 (1:500; clone EPR10376, ab174309, Abcam), rabbit anti-SCN4B (1:1000; ab80539, Abcam), mouse anti-polyglutamine expansion diseases marker (1:2000; clone 5TF1–1C2, MAB1574, EMD Millipore), and rabbit anti-vinculin (1:1000; clone E1E9V, #13901, Cell Signaling). Afterwards, blots were washed with TBS-T and incubated at room temperature for 1 h with the HRP-conjugated secondary antibody goat anti-rabbit (1:10,000; ab97051, Abcam) or with the respective secondary IRDye antibodies goat anti-mouse 680LT, goat anti-mouse 800CW, and goat anti-rabbit 800CW (all 1:10,000; LI-COR Biosciences). After final washing with TBS-T, chemiluminescence and fluorescence signals were detected using the LI-COR ODYSSEY^®^ FC and quantified with Image Studio 4.0 software (both LI-COR Biosciences).

### Bioinformatics

#### Quality control, alignment, and expression analysis

Read quality of RNA-seq data in fastq files was assessed using *FastQC* (v0.11.4)^75^ to identify sequencing cycles with low average quality, adaptor contamination, or repetitive sequences from PCR amplification. Reads were aligned using *STAR* (v2.5.2b)^76^ allowing gapped alignments to account for splicing against a custom-built genome composed of the *Ensembl Rattus norvegicus* genome v87 and the human *HTT* transgene. Alignment quality was analyzed using *samtools* (v1.1)^77^ and visually inspected in the *Integrative Genome Viewer* (v2.3.67)^78^. *Msantd1* was manually excluded from further downstream analyses as its differential expression likely results from its genomic sequence being present in the 50 kb 3′ flanking region of the transgenic *HTT* BAC construct. Normalized read counts for all genes were obtained using *DESeq2* (v1.16.1)^79^. Transcripts covered with less than 50 reads were excluded from the analysis leaving 12,639 genes for determining differential expression in each of the pair-wise comparisons between experimental groups.

The 2 × 3 factorial design of the experiment was captured in a generalized linear model describing expression (t) as a function of genotype (g), the environment (e), and their interaction (g × e). Surrogate variable analysis (*sva*, v3.24.4) was applied to minimize unwanted variation between samples^80^. Given that differences in transcript abundances in brain tissue are often small in magnitude and *in vivo* RNA-seq data are deemed to be more variable^81^, we set |*log*_*2*_ fold-change |≥0.3 and adjusted *p*-value ≤ 0.1 to determine differential expression, as computationally predicted candidates down to the lower end of these thresholds could be confirmed in qPCR assays.

Gene-level abundances were derived from *DESeq2* as normalized read counts and used for calculating the *log*_*2*_-transformed expression changes underlying the *k*-means clustering with ratios computed relative to the mean expression in WT_SE_. Raw counts provided by *DESeq2* also went into calculating nRPKMs (normalized Reads Per Kilobase per Million total reads) as a measure of relative gene expression as motivated before^82^. The *DESeq2 sizeFactors* served in scaling estimated abundances derived from *Salmon* (v0.7.2)^83^ when determining transcript-level compositions of individual genes. Transcript-level differential expression was further explored and verified with *kallisto* (v0.43.0)^84^.

#### Gene annotation, cell type enrichments, and pathway analyses

*ClusterProfiler* (v3.4.4) was employed to identify overrepresented Gene Ontology terms and associated cellular functions in sets of differentially expressed genes (DEGs)^85^. Terms with at least three annotated genes were queried and reported with their adjusted *p*-value ≤ 0.1. All Gene ID conversions were done using *biomaRt* Bioconductor package (v2.32.1) querying v87 of the *Ensembl* database.

Canonical pathways between sets of DEGs as well as predicted relationships between DEGs and disease aspects/biofunctions were derived from *Ingenuity Pathway Analysis* (IPA, v01–12, Qiagen). For the latter analysis module, only functions with at least three associated genes and at least one activating/inhibiting relationship are reported.

Expression data for 12-month-old animals are based on data files provided with Yu-Taeger *et al*.^12^. Cell type-specific attributions are based on RNA-seq data derived with Laser Capture Microdissection from main cellular populations of the striatum isolated from transgenic mice expressing eGFP in astrocytes (*Glt1*-eGFP^86^), microglia (*Cx3cr1*-eGFP^87^), *Drd2* medium spiny neurons (*Drd2*-eGFP^88^), and Tomato fluorescent protein in *Drd1* medium spiny neurons (*Drd1*-Tomato^89^) (unpublished data, Déglon lab). Enrichments of DEGs were calculated against 13 striatal consensus modules defined in Langfelder *et al*.^11^, of which 5 modules contain down- and 8 modules upregulated genes.

### Statistical analysis

Body weight data were analyzed in a three-way ANOVA with *Graph Pad Prism* (v7) using genotype as within-subject and age as well as environmental treatment as between-subject factors. Alpha was set at 0.05. ANOVA analyses were performed separately for the EE and MS because of different time spans of the treatments. SE groups served as control for both treatment conditions. Body weight results are shown in Supplementary Fig. S2.

For RT-qPCR data, two-way ANOVA was applied to test for genotype, environment, and their interaction followed by Tukey’s correction for multiple comparisons. Expression differences in the 12-month-old cohorts were tested with unpaired two-tailed *t*-test using significance thresholds of *p* < 0.05.

For Western blot data for *NR1D1*, *mGluR4,* and *SCN4B*, two-way ANOVA was applied to test for genotype, environment, and their interaction followed by Tukey’s correction for multiple comparisons. Gene expression differences in RT-qPCR data in the 12-month-old cohorts as well as EE and MS effects on *mHTT* protein expression in Western blot data were tested with unpaired two-tailed *t*-test using significance thresholds of *p* < 0.05.

## Electronic supplementary material

Supplementary Figures

Supplementary Table 1

## References

[1] Bates GP (2005). History of genetic disease: the molecular genetics of Huntington disease - a history. Nat Rev Genet.

[2] Waldvogel HJ, Kim EH, Tippett LJ, Vonsattel JP, Faull RL (2015). The Neuropathology of Huntington’s Disease. Curr Top Behav Neurosci.

[3] Vonsattel JP (1985). Neuropathological classification of Huntington’s disease. J Neuropathol Exp Neurol.

[4] Vonsattel JP, DiFiglia M (1998). Huntington disease. J Neuropathol Exp Neurol.

[5] Kumar A, Vaish M, Ratan RR (2014). Transcriptional dysregulation in Huntington’s disease: a failure of adaptive transcriptional homeostasis. Drug Discov Today.

[6] Cha JH (2007). Transcriptional signatures in Huntington’s disease. Prog Neurobiol.

[7] Thomas EA (2011). *In vivo* cell-autonomous transcriptional abnormalities revealed in mice expressing mutant huntingtin in striatal but not cortical neurons. Hum Mol Genet.

[8] Kuhn A (2007). Mutant huntingtin’s effects on striatal gene expression in mice recapitulate changes observed in human Huntington’s disease brain and do not differ with mutant huntingtin length or wild-type huntingtin dosage. Hum Mol Genet.

[9] Benn CL (2010). Environmental enrichment reduces neuronal intranuclear inclusion load but has no effect on messenger RNA expression in a mouse model of Huntington disease. J Neuropathol Exp Neurol.

[10] Becanovic K (2010). Transcriptional changes in Huntington disease identified using genome-wide expression profiling and cross-platform analysis. Hum Mol Genet.

[11] Langfelder P (2016). Integrated genomics and proteomics define huntingtin CAG length-dependent networks in mice. Nat Neurosci.

[12] Yu-Taeger L, Bonin M, Stricker-Shaver J, Riess O, Nguyen HH (2017). Dysregulation of gene expression in the striatum of BACHD rats expressing full-length mutant huntingtin and associated abnormalities on molecular and protein levels. Neuropharmacology.

[13] Reitz C, Mayeux R (2014). Alzheimer disease: epidemiology, diagnostic criteria, risk factors and biomarkers. Biochem Pharmacol.

[14] Ingre C, Roos PM, Piehl F, Kamel F, Fang F (2015). Risk factors for amyotrophic lateral sclerosis. Clin Epidemiol.

[15] Ascherio A, Schwarzschild MA (2016). The epidemiology of Parkinson’s disease: risk factors and prevention. Lancet Neurol.

[16] Mo C, Hannan AJ, Renoir T (2015). Environmental factors as modulators of neurodegeneration: insights from gene-environment interactions in Huntington’s disease. Neurosci Biobehav Rev.

[17] Zajac MS (2010). Wheel running and environmental enrichment differentially modify exon-specific BDNF expression in the hippocampus of wild-type and pre-motor symptomatic male and female Huntington’s disease mice. Hippocampus.

[18] Spires TL (2004). Environmental enrichment rescues protein deficits in a mouse model of Huntington’s disease, indicating a possible disease mechanism. J Neurosci.

[19] Pang TY, Du X, Zajac MS, Howard ML, Hannan AJ (2009). Altered serotonin receptor expression is associated with depression-related behavior in the R6/1 transgenic mouse model of Huntington’s disease. Hum Mol Genet.

[20] Nithianantharajah J, Barkus C, Murphy M, Hannan AJ (2008). Gene-environment interactions modulating cognitive function and molecular correlates of synaptic plasticity in Huntington’s disease transgenic mice. Neurobiol Dis.

[21] Lazic SE (2006). Neurogenesis in the R6/1 transgenic mouse model of Huntington’s disease: effects of environmental enrichment. Eur J Neurosci.

[22] Wood NI (2010). Responses to environmental enrichment differ with sex and genotype in a transgenic mouse model of Huntington’s disease. PLoS One.

[23] van Dellen A, Blakemore C, Deacon R, York D, Hannan AJ (2000). Delaying the onset of Huntington’s in mice. Nature.

[24] Renoir T (2013). Differential effects of early environmental enrichment on emotionality related behaviours in Huntington’s disease transgenic mice. J Physiol.

[25] Mo C, Renoir T, Pang TY, Hannan AJ (2013). Short-term memory acquisition in female Huntington’s disease mice is vulnerable to acute stress. Behav Brain Res.

[26] Wood NI, Glynn D, Morton AJ (2011). “Brain training” improves cognitive performance and survival in a transgenic mouse model of Huntington’s disease. Neurobiol Dis.

[27] Mo C, Renoir T, Hannan AJ (2014). Effects of chronic stress on the onset and progression of Huntington’s disease in transgenic mice. Neurobiol Dis.

[28] Mo C (2014). High stress hormone levels accelerate the onset of memory deficits in male Huntington’s disease mice. Neurobiol Dis.

[29] Yu-Taeger L (2012). A novel BACHD transgenic rat exhibits characteristic neuropathological features of Huntington disease. J Neurosci.

[30] Adjeroud N (2015). Reduced impact of emotion on choice behavior in presymptomatic BACHD rats, a transgenic rodent model for Huntington Disease. Neurobiol Learn Mem.

[31] Abada YS, Nguyen HP, Schreiber R, Ellenbroek B (2013). Assessment of motor function, sensory motor gating and recognition memory in a novel BACHD transgenic rat model for huntington disease. PLoS One.

[32] Abada YS, Nguyen HP, Ellenbroek B, Schreiber R (2013). Reversal learning and associative memory impairments in a BACHD rat model for Huntington disease. PLoS One.

[33] Pardon MC, Rattray I (2008). What do we know about the long-term consequences of stress on ageing and the progression of age-related neurodegenerative disorders?. Neurosci Biobehav Rev.

[34] de Kloet ER, Sibug RM, Helmerhorst FM, Schmidt MV (2005). Stress, genes and the mechanism of programming the brain for later life. Neurosci Biobehav Rev.

[35] Sale A, Berardi N, Maffei L (2014). Environment and brain plasticity: towards an endogenous pharmacotherapy. Physiol Rev.

[36] van Praag H, Kempermann G, Gage FH (2000). Neural consequences of environmental enrichment. Nat Rev Neurosci.

[37] Chen JY, Wang EA, Cepeda C, Levine MS (2013). Dopamine imbalance in Huntington’s disease: a mechanism for the lack of behavioral flexibility. Front Neurosci.

[38] Papale LA, Madrid A, Li S, Alisch RS (2017). Early-life stress links 5-hydroxymethylcytosine to anxiety-related behaviors. Epigenetics.

[39] Granholm L (2017). The expression of opioid genes in non-classical reward areas depends on early life conditions and ethanol intake. Brain Res.

[40] Smith R, Brundin P, Li JY (2005). Synaptic dysfunction in Huntington’s disease: a new perspective. Cell Mol Life Sci.

[41] Rozas JL, Gomez-Sanchez L, Tomas-Zapico C, Lucas JJ, Fernandez-Chacon R (2010). Presynaptic dysfunction in Huntington’s disease. Biochem Soc Trans.

[42] Brandstaetter H, Kruppa AJ, Buss F (2014). Huntingtin is required for ER-to-Golgi transport and for secretory vesicle fusion at the plasma membrane. Dis Model Mech.

[43] Woitecki AM (2016). Identification of Synaptotagmin 10 as Effector of NPAS4-Mediated Protection from Excitotoxic Neurodegeneration. J Neurosci.

[44] Ehrlich ME (2012). Huntington’s disease and the striatal medium spiny neuron: cell-autonomous and non-cell-autonomous mechanisms of disease. Neurotherapeutics.

[45] Jiang R, Diaz-Castro B, Looger LL, Khakh BS (2016). Dysfunctional Calcium and Glutamate Signaling in Striatal Astrocytes from Huntington’s Disease Model Mice. J Neurosci.

[46] Bradford J (2010). Mutant huntingtin in glial cells exacerbates neurological symptoms of Huntington disease mice. J Biol Chem.

[47] Oyama F (2006). Sodium channel beta4 subunit: down-regulation and possible involvement in neuritic degeneration in Huntington’s disease transgenic mice. J Neurochem.

[48] Ribeiro FM (2014). Metabotropic glutamate receptor 5 knockout promotes motor and biochemical alterations in a mouse model of Huntington’s disease. Hum Mol Genet.

[49] Buschler A, Manahan-Vaughan D (2017). Metabotropic glutamate receptor, mGlu5, mediates enhancements of hippocampal long-term potentiation after environmental enrichment in young and old mice. Neuropharmacology.

[50] Flor PJ, Battaglia G, Nicoletti F, Gasparini F, Bruno V (2002). Neuroprotective activity of metabotropic glutamate receptor ligands. Adv Exp Med Biol.

[51] Sun H, Kosaras B, Klein PM, Jensen FE (2013). Mammalian target of rapamycin complex 1 activation negatively regulates Polo-like kinase 2-mediated homeostatic compensation following neonatal seizures. Proc Natl Acad Sci USA.

[52] Du X (2015). The influence of the HPG axis on stress response and depressive-like behaviour in a transgenic mouse model of Huntington’s disease. Exp Neurol.

[53] Aziz NA (2009). Increased hypothalamic-pituitary-adrenal axis activity in Huntington’s disease. J Clin Endocrinol Metab.

[54] Iwasaki S, Inoue K, Kiriike N, Hikiji K (2000). Effect of maternal separation on feeding behavior of rats in later life. Physiol Behav.

[55] Lenglos C, Mitra A, Guevremont G, Timofeeva E (2013). Sex differences in the effects of chronic stress and food restriction on body weight gain and brain expression of CRF and relaxin-3 in rats. Genes Brain Behav.

[56] Harris RB (2002). Weight loss in rats exposed to repeated acute restraint stress is independent of energy or leptin status. Am J Physiol Regul Integr Comp Physiol.

[57] Marais L, van Rensburg SJ, van Zyl JM, Stein DJ, Daniels WM (2008). Maternal separation of rat pups increases the risk of developing depressive-like behavior after subsequent chronic stress by altering corticosterone and neurotrophin levels in the hippocampus. Neurosci Res.

[58] Hulshof HJ (2011). Maternal separation decreases adult hippocampal cell proliferation and impairs cognitive performance but has little effect on stress sensitivity and anxiety in adult Wistar rats. Behav Brain Res.

[59] Wigger A, Neumann ID (1999). Periodic maternal deprivation induces gender-dependent alterations in behavioral and neuroendocrine responses to emotional stress in adult rats. Physiol Behav.

[60] Plotsky PM (2005). Long-term consequences of neonatal rearing on central corticotropin-releasing factor systems in adult male rat offspring. Neuropsychopharmacology.

[61] Plotsky PM, Meaney MJ (1993). Early, postnatal experience alters hypothalamic corticotropin-releasing factor (CRF) mRNA, median eminence CRF content and stress-induced release in adult rats. Brain Res Mol Brain Res.

[62] Rubin TG, Gray JD, McEwen BS (2014). Experience and the ever-changing brain: what the transcriptome can reveal. Bioessays.

[63] Manfre G (2017). The BACHD Rat Model of Huntington Disease Shows Specific Deficits in a Test Battery of Motor Function. Front Behav Neurosci.

[64] Clemensson EK, Clemensson LE, Fabry B, Riess O, Nguyen HP (2017). Further investigation of phenotypes and confounding factors of progressive ratio performance and feeding behavior in the BACHD rat model of Huntington disease. PLoS One.

[65] Novak G, Fan T, O’Dowd BF, George SR (2013). Striatal development involves a switch in gene expression networks, followed by a myelination event: implications for neuropsychiatric disease. Synapse.

[66] Solt LA (2012). Regulation of circadian behaviour and metabolism by synthetic REV-ERB agonists. Nature.

[67] Maier T, Guell M, Serrano L (2009). Correlation of mRNA and protein in complex biological samples. FEBS Lett.

[68] Williams CJ, Dexter DT (2014). Neuroprotective and symptomatic effects of targeting group III mGlu receptors in neurodegenerative disease. J Neurochem.

[69] Betts MJ, O’Neill MJ, Duty S (2012). Allosteric modulation of the group III mGlu4 receptor provides functional neuroprotection in the 6-hydroxydopamine rat model of Parkinson’s disease. Br J Pharmacol.

[70] Slotten HA, Kalinichev M, Hagan JJ, Marsden CA, Fone KC (2006). Long-lasting changes in behavioural and neuroendocrine indices in the rat following neonatal maternal separation: gender-dependent effects. Brain Res.

[71] Wang Q, Shao F, Wang W (2015). Maternal separation produces alterations of forebrain brain-derived neurotrophic factor expression in differently aged rats. Front Mol Neurosci.

[72] Pfaffl MW (2001). A new mathematical model for relative quantification in real-time RT-PCR. Nucleic Acids Res.

[73] Andersen CL, Jensen JL, Orntoft TF (2004). Normalization of real-time quantitative reverse transcription-PCR data: a model-based variance estimation approach to identify genes suited for normalization, applied to bladder and colon cancer data sets. Cancer Res.

[74] Vandesompele J (2002). Accurate normalization of real-time quantitative RT-PCR data by geometric averaging of multiple internal control genes. Genome Biol.

[75] Andrews, S. *FastQC: a quality control tool for high throughput sequence data*. http://www.bioinformatics.babraham.ac.uk/projects/fastqc (2010).

[76] Dobin A (2013). STAR: ultrafast universal RNA-seq aligner. Bioinformatics.

[77] Li H (2009). The Sequence Alignment/Map format and SAMtools. Bioinformatics.

[78] Thorvaldsdottir H, Robinson JT, Mesirov JP (2013). Integrative Genomics Viewer (IGV): high-performance genomics data visualization and exploration. Brief Bioinform.

[79] Love MI, Huber W, Anders S (2014). Moderated estimation of fold change and dispersion for RNA-seq data with DESeq2. Genome Biol.

[80] Leek JT, Johnson WE, Parker HS, Jaffe AE, Storey JD (2012). The sva package for removing batch effects and other unwanted variation in high-throughput experiments. Bioinformatics.

[81] Maze I (2014). Analytical tools and current challenges in the modern era of neuroepigenomics. Nat Neurosci.

[82] Srinivasan K (2016). Untangling the brain’s neuroinflammatory and neurodegenerative transcriptional responses. Nat Commun.

[83] Patro R, Duggal G, Love MI, Irizarry RA, Kingsford C (2017). Salmon provides fast and bias-aware quantification of transcript expression. Nat Methods.

[84] Bray NL, Pimentel H, Melsted P, Pachter L (2016). Near-optimal probabilistic RNA-seq quantification. Nat Biotechnol.

[85] Yu G, Wang LG, Han Y, He Q (2012). Y. clusterProfiler: an R package for comparing biological themes among gene clusters. OMICS.

[86] Regan MR (2007). Variations in promoter activity reveal a differential expression and physiology of glutamate transporters by glia in the developing and mature CNS. J Neurosci.

[87] Jung S (2000). Analysis of fractalkine receptor CX(3)CR1 function by targeted deletion and green fluorescent protein reporter gene insertion. Mol Cell Biol.

[88] Gong S (2003). A gene expression atlas of the central nervous system based on bacterial artificial chromosomes. Nature.

[89] Ade KK, Wan Y, Chen M, Gloss B, Calakos N (2011). An Improved BAC Transgenic Fluorescent Reporter Line for Sensitive and Specific Identification of Striatonigral Medium Spiny Neurons. Front Syst Neurosci.

