# Supplementary material for: Environment-dependent striatal gene expression in the BACHD rat model for Huntington disease
Source: Sci Rep. 2018 Apr 11;8:5803. doi: 10.1038/s41598-018-24243-z (PMC5895842; doi:10.1038/s41598-018-24243-z)
Supplement: Supplementary file 1 — Supplementary Figures [file 41598_2018_24243_MOESM1_ESM.pdf]

SUPPLEMENTARY INFORMATION

Environment-dependent striatal gene expression in the BACHD rat model for Huntington disease

Arianna Novati[1*], Thomas Hentrich[1*], Zinah Wassouf[1], Jonasz J. Weber[1], Libo Yu-Taeger[1], Nicole Déglon[2], Huu Phuc Nguyen[1+] and Julia M. Schulze-Hentrich[1+]

1 Institute of Medical Genetics and Applied Genomics, University of Tübingen, Tübingen, Germany.

2 Department of Clinical Neurosciences (DNC), Lausanne University Hospital (CHUV), Lausanne, Switzerland.

[*], [+] These authors contributed equally to this work.

Supplementary Figure 1

a

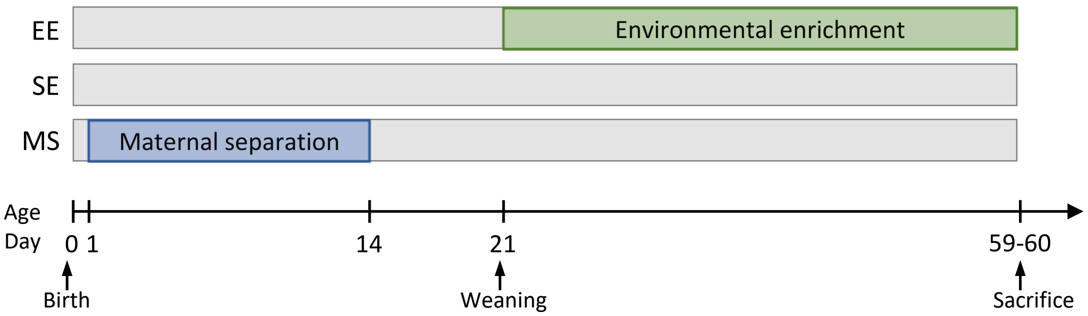

b

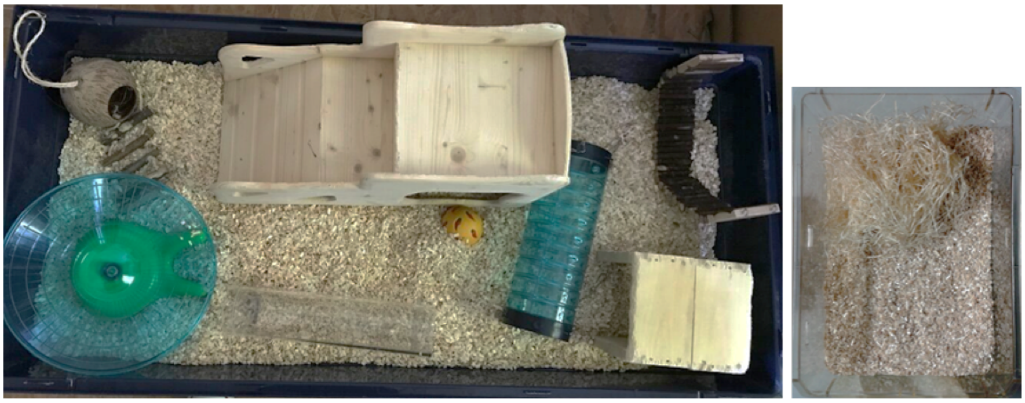

Supplementary Fig. 1. Experimental timeline and housing conditions.

(a) Experimental groups of WT and BACHD rats were allocated to three treatment paradigms: SE, EE, and MS. Day of birth indicated as PD 0. All animals in EE and SE groups were kept in standard environment until weaning and then housed in either the enriched or standard environment to an age of 59-60 days, respectively. Animals in the MS groups were repeatedly separated from their mothers for 4 h daily during P1-P14 and kept in standard environment after weaning to an age of 59-60 days.

(b) Left panel shows layout of an EE cage with toys repeatedly rearranged. Right panel for size comparisons between EE and SE cages. The latter contained bedding and nesting material only.

Supplementary Figure 2

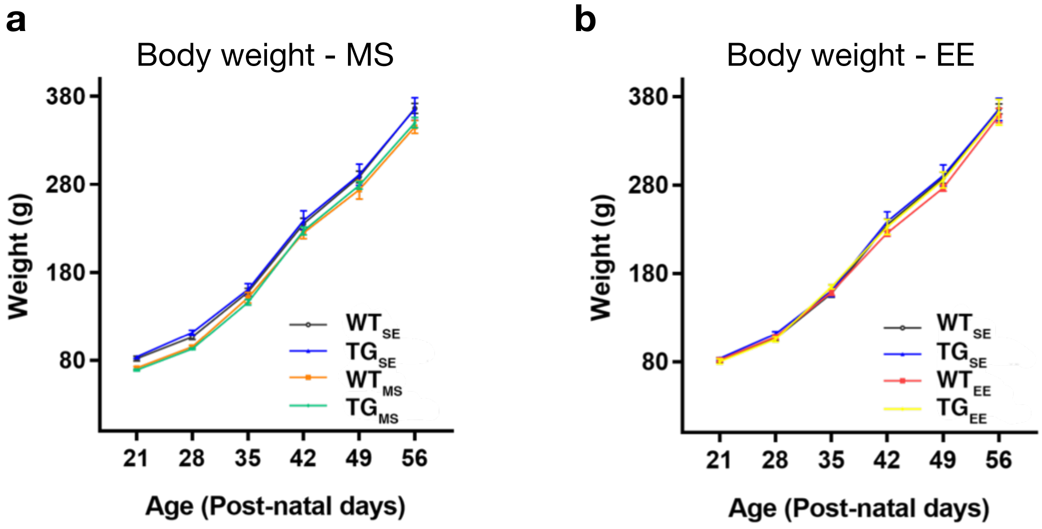

Supplementary Fig. 2. Body weight development in BACHD rats for tested environments.

Body weight development of WT and BACHD (TG) rats in MS (a) and EE (b) from weaning (PD 21) till PD 56. Animals in all experimental groups increased body weight during the period of the experimental procedure. Three way ANOVA: age effect for MS / SE: $F_{(5, 168)} = 1190$; $p < 0.0001$; for EE / SE: $F_{(5, 168)} = 996.9$; $p < 0.0001$. MS-animals weighted less than control animals in SE (a) ($F_{(1, 168)} = 27.48$; $p < 0.0001$). EE did not significantly affect body weight (b). No significant change in body weight dependent on genotype was observed in any of the treatment conditions. Data presented as mean (±SEM) with n = 8 per group.

Supplementary Figure 3

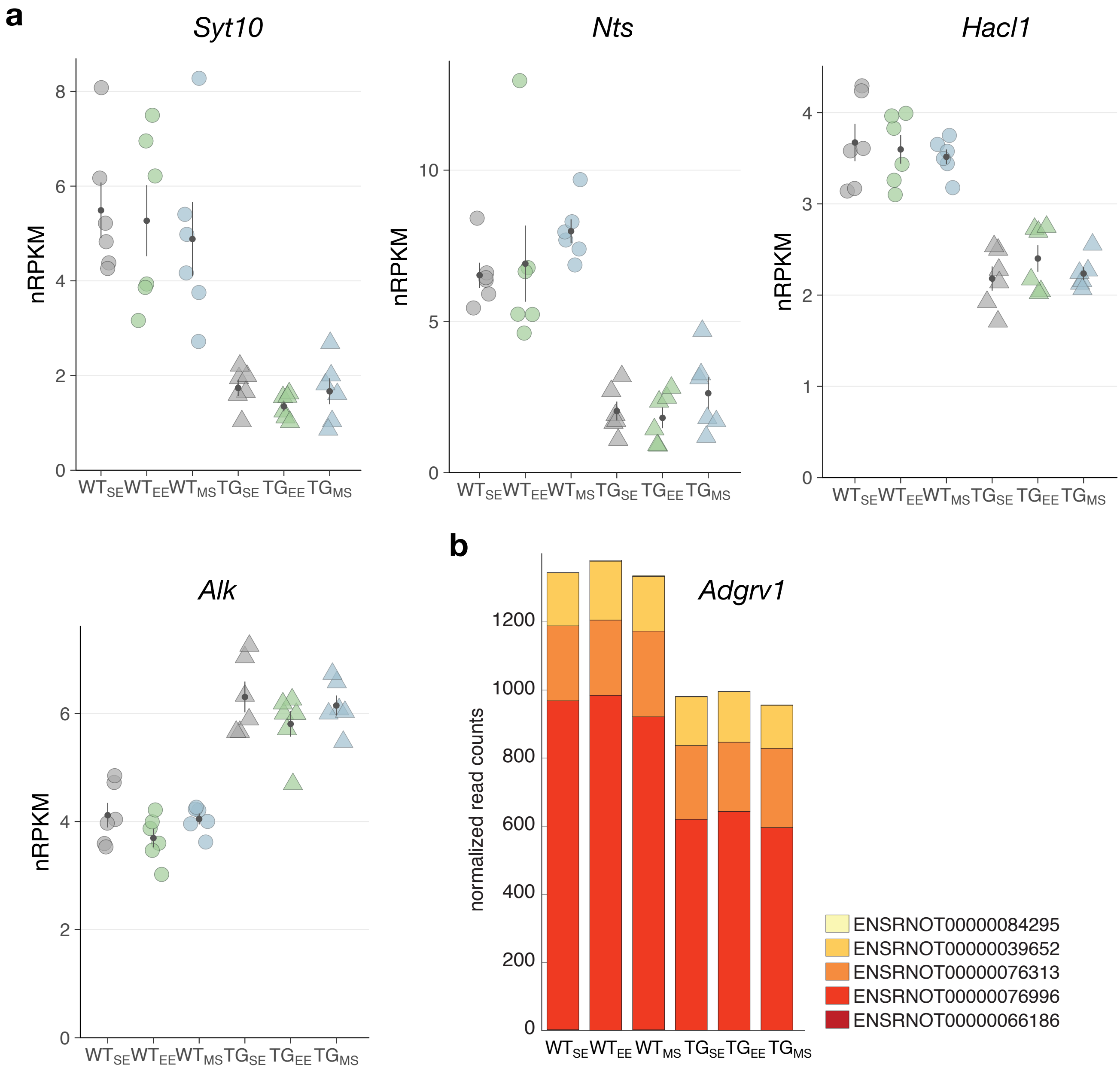

Supplementary Fig. 3. DEGs similarly disturbed in BACHD rats across three environmental conditions.

(a) Expression levels of DEGs differentially expressed in BACHD rats under three environmental conditions plotted as individual data points with mean ± SEM.

(b) Composition and expression level of *Adgrv1* splice variants across experimental groups.

Supplementary Figure 4

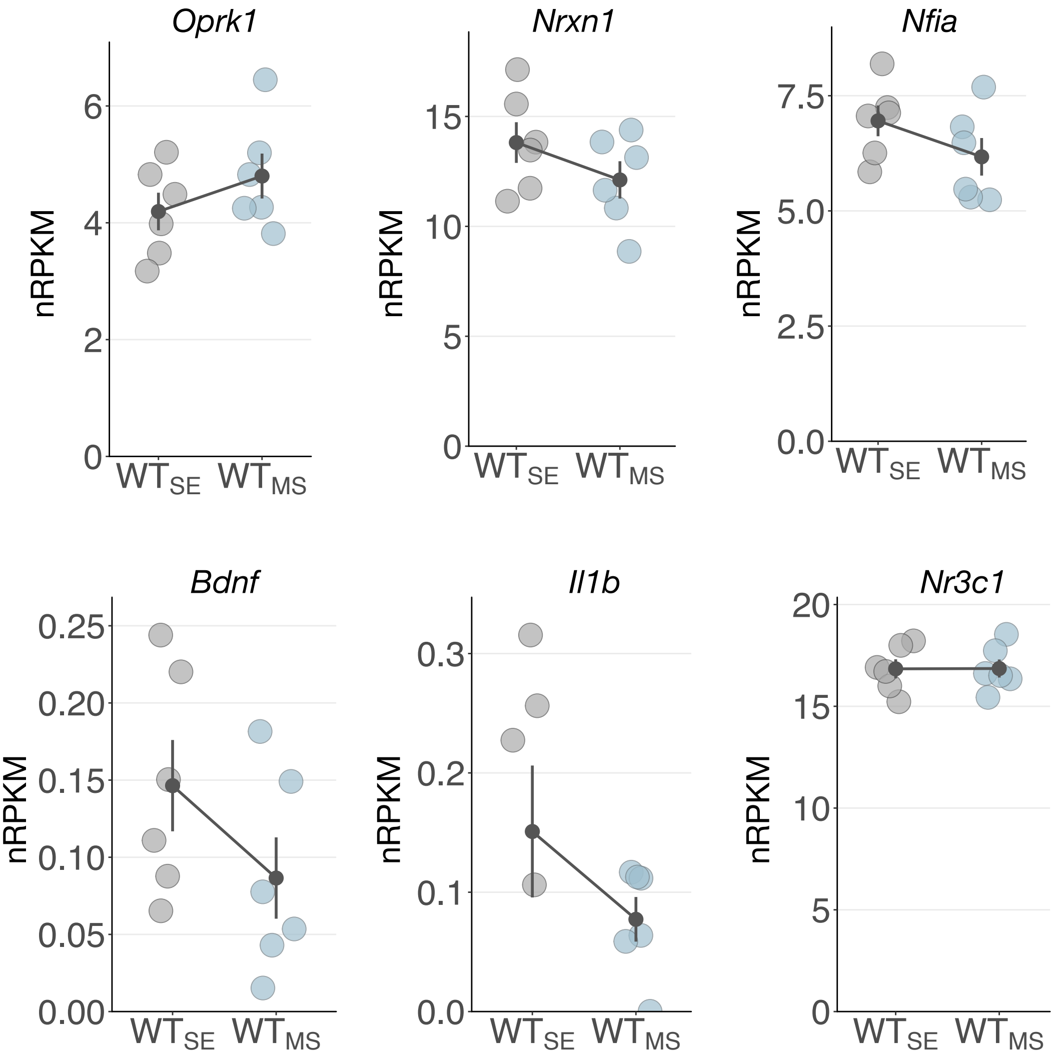

Supplementary Fig. 4. Selected stress-response genes showed trends of changed expression upon maternal separation.

Expression levels of selected stress-response genes for WT rats after MS compared to SE plotted as individual data points with mean ± SEM.

Supplementary Figure 5

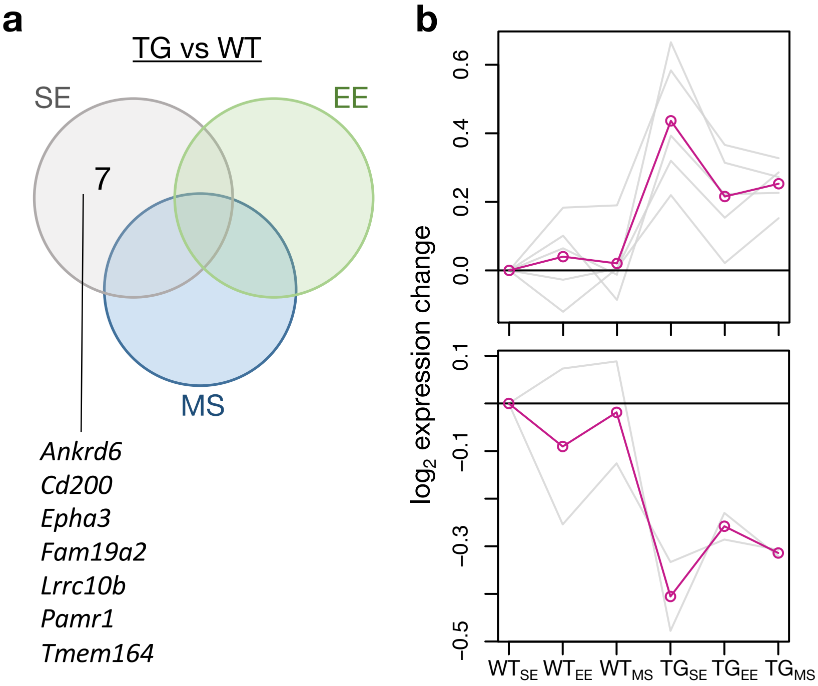

Supplementary Fig. 5. DEGs responding similarly to EE and MS in BACHD rats.

(a) Venn diagram highlighting seven DEGs identified in SE which were neither differentially expressed in EE nor MS.

(b) Clustering seven DEGs in (a) using *k*-means on gene expression ratios relative to WT_{SE} across four experimental groups. Grey lines indicate expression changes per gene, pink lines cluster centroids.

Supplementary Figure 6

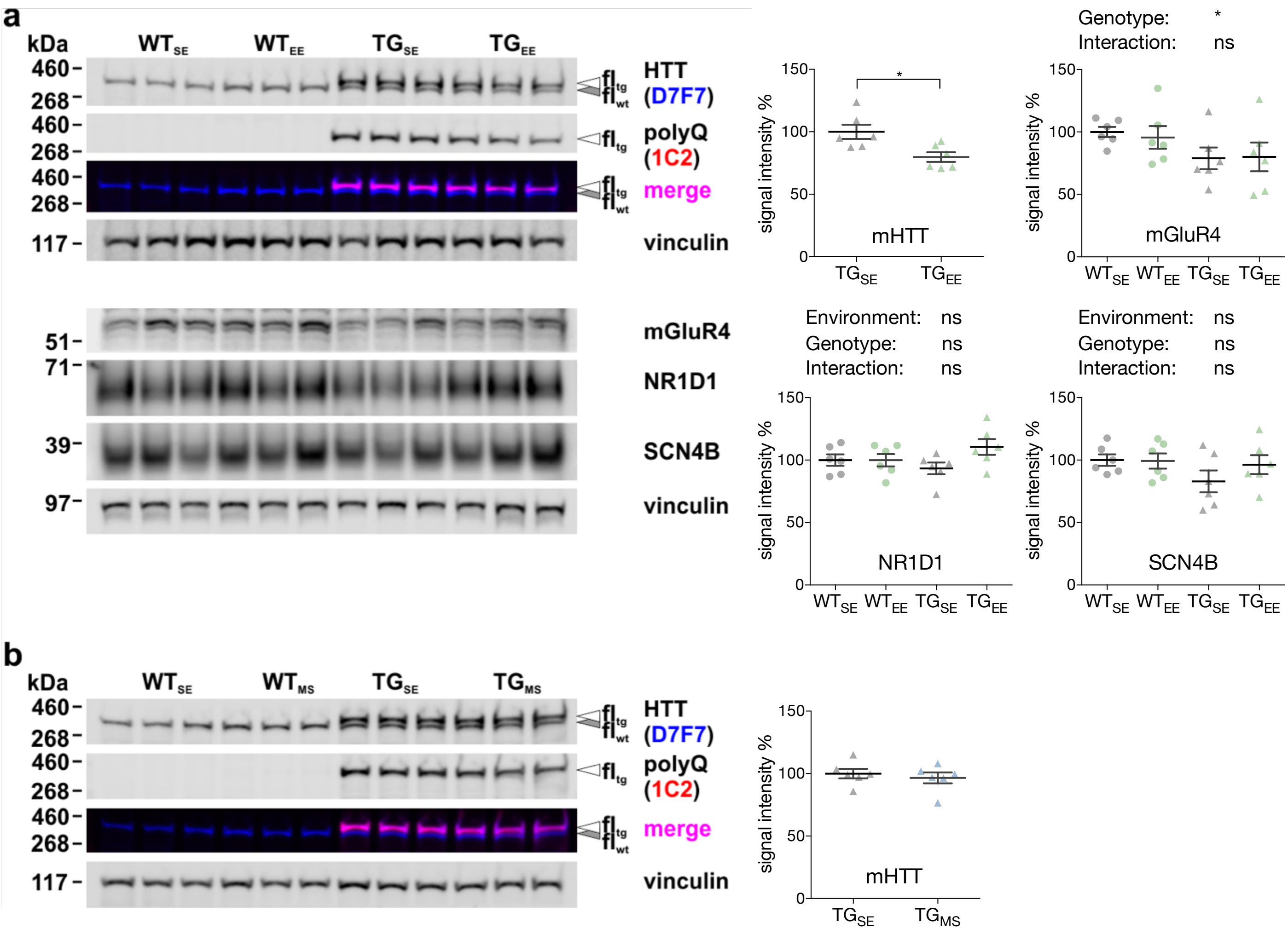

Supplementary Fig. 6.

(a) Representative protein blots of wildtype *HTT*, mutant *HTT*, *mGluR4*, *NR1D1* and *SCN4B* detected in striatal homogenates across SE and EE groups (n = 6 rats per group). Vinculin was used for normalization. Quantifications relative to WT_{SE} (or TG_{SE} for *mHTT*) are plotted as mean ± SEM. Two-way ANOVA followed by Tukey's multiple comparisons test, and unpaired two-tailed *t*-test for the difference in mHTT were performed $*p < 0.05$. White arrowheads = mutant *HTT* (*mHTT*); grey arrowhead = endogenous rat *HTT*.

(b) Representative protein blots of wildtype and mutant *HTT* in striatal homogenates across SE and MS groups. Vinculin was used for normalization (n = 6 rats per group).

Supplementary Table S1. Primer list

Primers used for RT-qPCR validation of RNA-seq results.

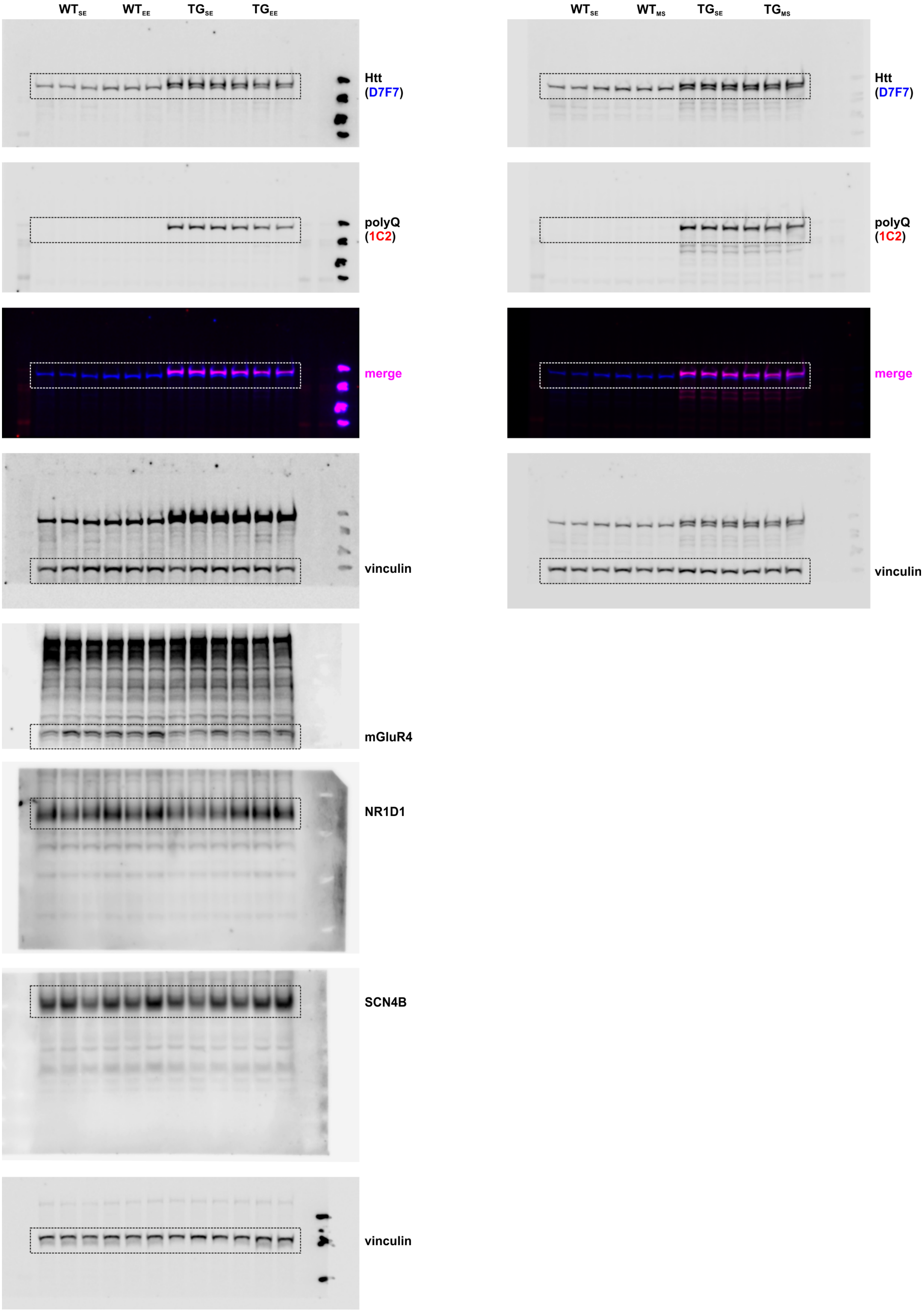
WT$_{SE}$ WT$_{EE}$ TG$_{SE}$ TG$_{EE}$
Htt (D7F7)
polyQ (1C2)
merge
vinculin
mGluR4
NR1D1
SCN4B
vinculin
WT$_{SE}$ WT$_{MS}$ TG$_{SE}$ TG$_{MS}$
Htt (D7F7)
polyQ (1C2)
merge
vinculin
